# Research on the effects of rs1800566 C/T polymorphism of NAD(P)H quinone oxidoreductase 1 gene on cancer risk involves analysis of 43,736 cancer cases and 56,173 controls

**DOI:** 10.3389/fonc.2022.980897

**Published:** 2022-10-19

**Authors:** Hangsheng Zhou, Hongyuan Wan, Lijie Zhu, Yuanyuan Mi

**Affiliations:** ^1^ Wuxi Medical College, Jiangnan University, Wuxi, China; ^2^ Department of Urology, Affiliated Hospital of Jiangnan University, Wuxi, China

**Keywords:** cancer, NADPH quinone dehydrogenase-1 (NQO1), polymorphism, tumor marker, meta - analysis

## Abstract

**Objective:**

A two-electron reductase known as NQO1 [NAD(P)H quinone oxidoreductase 1] is regarded as an excellent anticancer target. Studies have found that rs1800566 polymorphism of NQO1 is linked to different cancers, but their associations remain controversial.

**Methods:**

In the present work, we selected to do a comprehensive meta-analysis to analyze their correlation. We performed searches on PubMed, Embase, Google Scholar, Chinese database, and Web of Science. The results we obtained covered all publications before April 3, 2022.

**Results:**

There were 176 case-control studies among them, with 56,173 corresponding controls and 43,736 cancer cases. We determined that the NQO1 rs1800566 polymorphism was not related to the cancer risk by calculating 95% confidence intervals and odds ratios. However, stratified genotyping showed that this polymorphism was protective against hepatocellular carcinoma, renal cell carcinoma, and gastric cancer. In addition, on dividing cancer into six systems, the association with gastrointestinal cancer decreased. In the race-based subgroup, a decreasing trend was observed in Asians, while an increasing trend was found among Caucasians, Africans, and mixed populations. The decreased correlation in the hospital-based subgroup was also detected.

**Conclusion:**

Current study shows that rs1800566 polymorphism of NQO1 was linked to cancer susceptibility and maybe as a tumor marker in their development.

## Introduction

Cancer is the leading cause of mortality globally, making it difficult to increase the average lifespan. According to 2019 statistics from the WHO, cancer is one of the major or secondary reasons behind death for people before age 70 in 112 of 183 countries (1). Global population growth and an increase in life expectancy led to an increase in cancer incidence and death rates in epidemiological statistics. It is influenced by social and economic development factors along with changes in the prevalence and carcinogenic factors distribution (2). Cancer is caused primarily by genetic and environmental factors (1).

One prevalent sequence variation type in the human genome is called a single nucleotide polymorphism (SNP) (3). SNPs are abundant in the human genome, numbering more than 10 million (4). The selective maintenance of point mutations in populations is an important reason for the occurrence of SNPs, and their frequency is determined by four factors, including the time after mutation, the evolutionary pressure associated with significant functional variations in organisms, and random genetic drift and bottleneck events (5). There are millions of SNPs in the human genome, most of which can’t alter gene expression or function. The main challenge is to select SNPs that may affect phenotypic function and ultimately lead to disease development (6). Recent studies showed that SNPs associated with cancer risk through affecting the expression levels of nearby genes (7). At present, the treatment of cancer is no longer limited to traditional surgeries, chemotherapy, radiotherapy, etc. Targeting actionable initiates in oncogene-driven cancer and immuno-oncology dramatically increases the diversity of cancer therapy (8). Based on above information, the research on SNPs of multiple genes may help to develop effective treatment.

NAD(P)H quinone oxidoreductase 1 (NQO1) is a two-electron reductase responsible for detoxification of quinones and also bioactivation of certain quinones. It is encoded by the NQO1 gene and is mapping to chromosomal location 16q22.1 (9). In a variety of tumors, NQO1 has been shown to be overexpressed and capable of bioactivating certain quinone substrates, which means it is an ideal target for cancer treatment (10). The association between NQO1 and the cancer suppressor p53 has been more deeply studied. NQO1 physically interacts with p53 and p73 in an NADH-dependent manner and protects them from 20S proteasome degradation (11). NQO1 and proteasome 20S have a feedback loop that inhibits each other. The rs1800566 polymorphism may reduce the level of NQO1 protein in cell lines and tissues of homozygous organisms, resulting that the tumor suppressor p53 is no longer protected, thus promoting tumor formation (12). NQO1 has been found to have many polymorphisms, among which the most studied have been focused on the rs1800566 site. The study found that patients who were CT carriers of NQO1 rs1800566 needed a 13% higher dose of warfarin than those who were CC carriers (13). Similarly, studies in cancer showed that rs1800566 polymorphism of NQO1 was an important risk factor for hepatocellular carcinoma (14). In another cancer study, the NQO1 rs1800566 polymorphism has been linked with poorer response to drugs of chemotherapy. Several studies have since reported links between rs1800566 polymorphisms and other cancer types. We performed a comprehensive analysis including 43,736 cancer cases and 56,173 controls genotyping of the rs1800566 polymorphism of NQO1 to assess the effect of this functional SNP on cancer susceptibility. Since the product of NQO1 gene expression is believed to exert universal antioxidant and cellular protective effects in tissues, our study will help to elucidate the effect and biological significance of this gene polymorphism on overall cancer risk. The NQO1 gene rs1800566 polymorphism has been widely discussed in the literature as a related risk factor in cancer. Although two previous meta-analyses (Guo (15) and Ding (16)) has been reported about NQO1 gene rs1800566 polymorphism, they focused on single cancer type, included a small samples, went short of comprehensive subgroups. Besides, in 2013, Lajin et al. performed a similar analysis involved 92 studies including 21178 cases and 25157 control, some limitations also existed: relatively small sample size, a small number of positive results (17). In the past ten years, a larger number of studies have been published, combined the vital role of this gene polymorphism, it is necessary to update this association. We wish to obtain some new discovery. Our present study was based on larger samples and multiple subgroup analysis, including its expression and overall survival analysis and meta-regression analysis.

## Materials and methods

### Finding and analyzing appropriate studies

Search terms for ‘NQO1’ or ‘NAD(P)H quinone reductase 1’, ‘cancer’ and ‘polymorphism,’ were used to scour Google Scholar, the Chinese database, Embase, Web of Science and PubMed (last updated: April 3, 2022). There were 15222 articles searched using these terms, and finally, 176 articles met the criteria. A manual search of references for the review articles was also conducted.

### Inclusion and exclusion criteria

In this review, cancer risk was evaluated, case-control studies were included, and each group had enough genotypes to be included in the analysis (cases and controls). We also excluded studies that (a) did not employ a control sample, (b) did not provide genotype frequency, or (c) were similar with those already published.

### Data extraction

Separately, two authors-based selection criteria were employed to retrieve these data. Data was collected on the surname of the first author, publication year, the cancer type, the country of origin, the control subject’s ethnicity, the number of controls and cases, the degree to which the control subjects were in the genotyping techniques, and Hardy Weinberg equilibrium (HWE) employed.

### Statistics analysis

Stratification by cancer type was the first step. There are two or more studies in each subgroup. Moreover, we grouped cancer into six systems: urinary system cancer, digestive cancer, gynecological cancer, hematological cancer, respiratory cancer, and head-neck cancer. Asian, African, Caucasian, and Mixed individuals were divided into four ethnic groups. According to their origins, an analysis of the hospital-based and population-based (PB) control subgroups was conducted. Based on the distributions of the genotypes of cases and controls, we determined odds ratios (OR) and 95% confidence intervals (CI) for the association between the NQO1 rs1800566 polymorphism and cancer risk. In order to examine the OR as a whole, a Z-test was performed (18). Heterogeneity was evaluated using chi-squared Q-tests, the P value was larger than 0.05, hence there was no indication of statistically significant heterogeneity across the trials. To account for potential considerable heterogeneity, we used the random-effects model; nonetheless, we relied on the fixed-effects approach (19, 20). Moreover, dominant genetic model (CC+CT vs. TT), recessive genetic model (CC vs. CT+TT), homozygote comparison (CC vs. TT), allelic contrast (C-allele vs. T-allele), and heterozygote comparison (CT vs. TT) were used to examine the relationship between the NQO1 rs1800566 cancer risk and genetic variation. The HWE in the control groups was calculated using Pearson’s chi-square test. Using the Egger regression test and Begg’s funnel plots, publication bias was calculated (21). Additionally, Stata software 11.0 (StataCorp LP, College Station, TX) was used to conduct the statistical analyses for our meta-analysis.

### Meta-regression

A random effect meta-regression analysis was used to determine the cause of the publishing bias; the independent variable was the publication year as a subgroup, race, control source, and genotype method, while the values of log was considered as the dependent variable (22).

### Bioinformatics analysis

GEPIA (http://gepia.cancer-pku.cn/) provides information about NQO1 expression in most types of cancer and adjacent tissues. In addition, statistics on NQO1 expression levels, such as disease-free and overall survival, are available.

## Results

### Meta-analysis study characteristics

**Figure 1:**
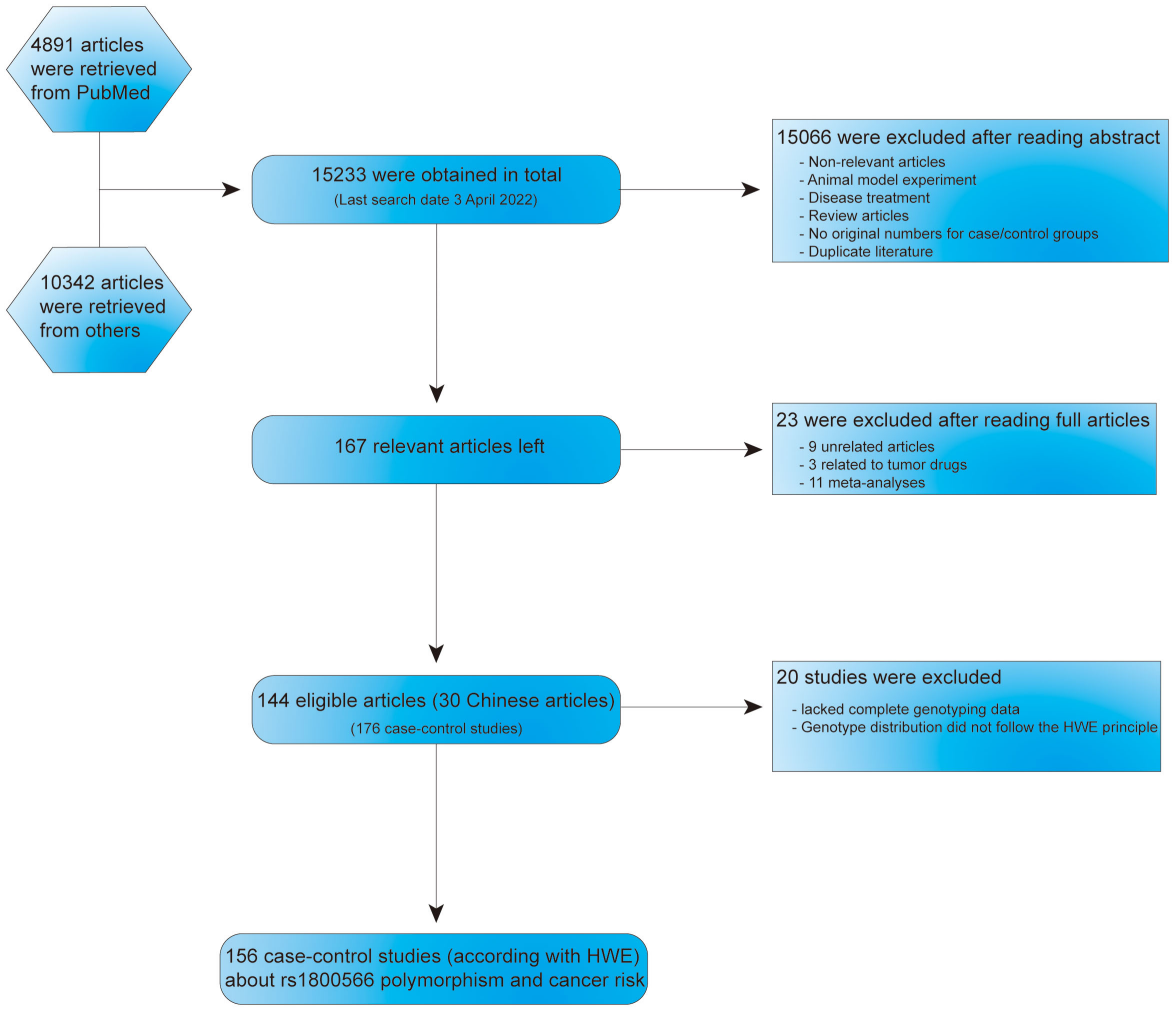
Flow chart about the search and screening strategies for NQO1 rs1800566 polymorphism studies from several database.

**Figure 2:**
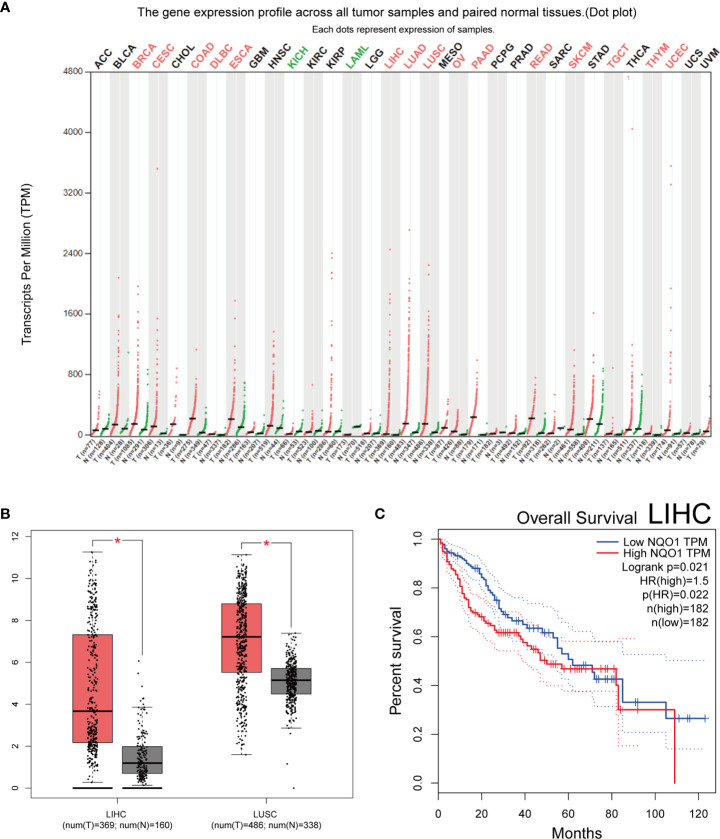
Bioinformatics study of the NQO1 gene. **(A)** profile of the NQO1 gene’s expression in each tumor sample and its matched normal tissues. The expressing of NQO1 in many different types of cancer (Red represents high expression, green represents low expression, and black represents no significantly difference). **(B)** NQO1 gene expression in both the LIHC and the LUSC. **(C)** Analysis of LIHC overall survival, the expression level of NQO1 was negatively correlated with the overall survival rate of patients with LIHC. ACC, adrenocortical carcinoma; BLCA, bladder urothelial carcinoma; BRCA, breast invasive carcinoma; CESC, cervical squamous cell carcinoma and endocervical adenocarcinoma; CHOL, cholangiocarcinoma; COAD, colon adenocarcinoma; DLBC, lymphoid neoplasm diffuse large B-cell lymphoma; ESCA, esophageal carcinoma; GBM, glioblastoma multiforme; HNSC, head and neck squamous cell carcinoma; KICH, kidney chromophobe; KIRC, kidney renal clear cell carcinoma; KIRP, kidney renal papillary cell carcinoma; LAML, acute myeloid leukemia; LGG, brain lower grade glioma; LIHC, liver hepatocellular carcinoma; LUAD, lung adenocarcinoma; LUSC, lung squamous cell carcinoma; MESO, mesothelioma; OV, ovarian serous cystadenocarcinoma; PAAD, pancreatic adenocarcinoma; PCPG, pheochromocytoma and paraganglioma; PRAD, prostate adenocarcinoma; READ, rectum adenocarcinoma; SARC, sarcoma; SKCM, skin cutaneous melanoma; STAD, stomach adenocarcinoma; TGCT, testicular germ cell tumors; THCA, thyroid carcinoma; THYM, thymoma; UCEC, uterine corpus endometrial carcinoma; UCS, uterine carcinosarcoma; UVM, uveal melanoma. *:p < 0.05.

Identification of multiple databases including 15233 articles, and after careful screening, we included 167 different articles (retrieved on April 3, 2022). Subsequently, we excluded nine unrelated articles, three articles related to tumor drugs and 11 meta-analyses. Then, 144 articles (176 case-control studies), including 30 Chinese language papers were confirmed. Among them, 20 case-control studies were deficient complete genotyping data or were not according with HWE principle. Finally, 156 case-control studies related to NQO1 rs1800566 polymorphism and cancer risk were obtained (**Figure 1**). In **Supplementary Table 1**, all information concerning the literature was presented, including first author, number of controls and cases, cancer type, year of publication, ethnicity, genotyping method, and control sources. Among the case-control studies retrieved, 43,736 cases and 56,173 controls were included, and the control group comprised mainly healthy people. Among them were 82 Asian, six African, 83 Caucasian, and five mixed population studies. One hundred and fifteen studies were based on HB source, while 61 were from PB. The minor allele frequency (MAF) of this locus in six major global populations were analyzed in 1000 Genomes Browser (https://www.ncbi.nlm.nih.gov/snp/rs1800566) (**Supplementary Figures 1A, B** **)**. To retrieve the polymorphism of rs1800566, we also applied the TCGA database and the results showed that TT(AA) frequency was the highest among the three genotypes (**Supplementary Figure 1C**). Cancers of the heart, lung, colon, prostate, and skin have all been associated to this polymorphism (https://www.gtexportal.org/home/) (**Supplementary Figure 1D**). In twenty studies, genotypes were not determined based on HWE. The expression of NQO1 was significantly different between cancer and normal tissues in seventeen kinds of tumors (**Figure 2A**). Among above, the expression of NQO1 was higher in cancer than normal tissue (P<0.05), such as liver hepatocellular carcinoma (LIHC) and lung squamous cell carcinoma (LUSC) (**Figure 2B**). In addition, higher expression of NQO1 in LIHC patients may had significantly poor overall survival from Kaplan-Meier (**Figure 2C**).

### Meta-analysis

**Figure 3:**
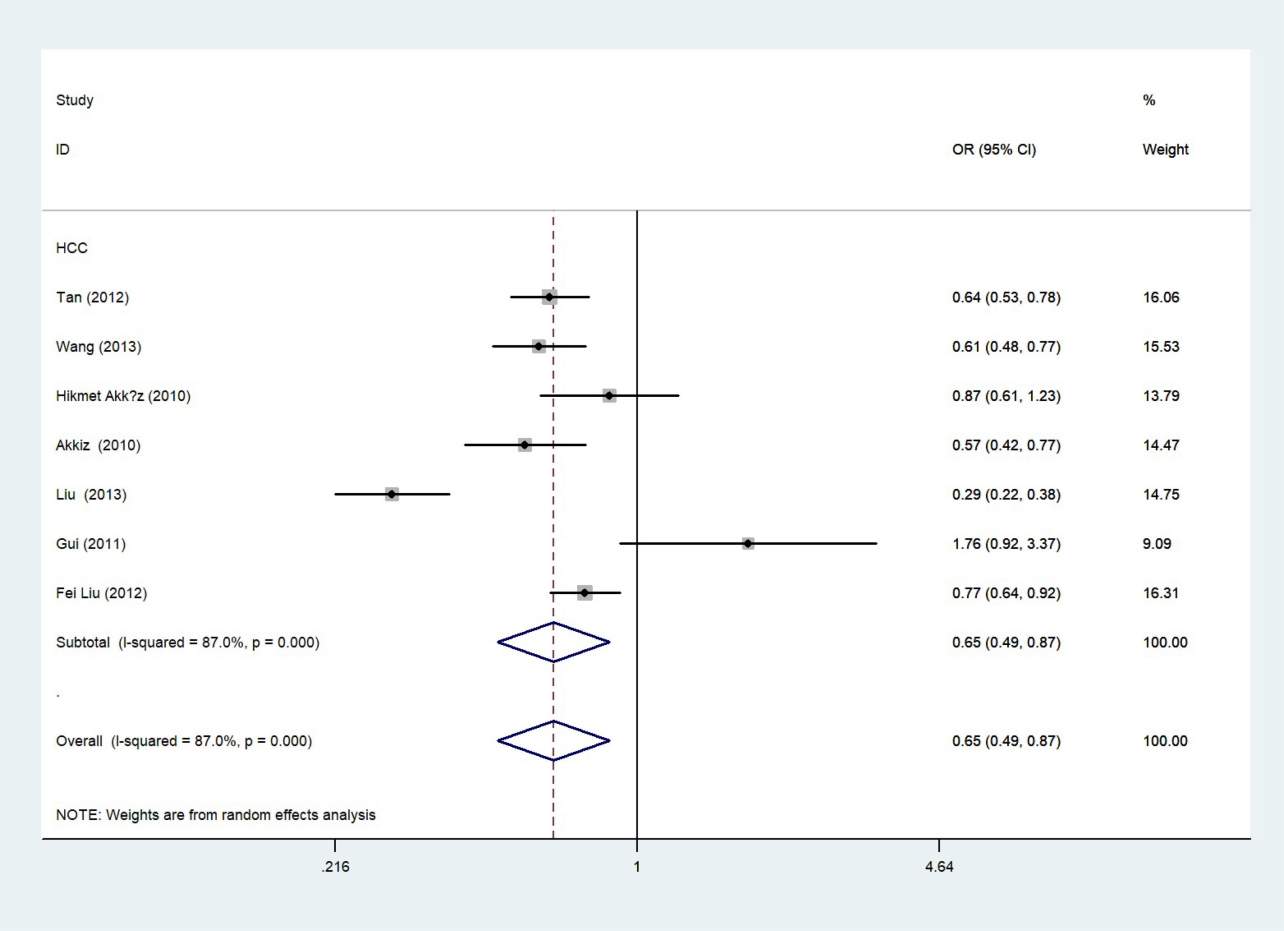
Forest plot about the relationship between the risk of HCC and the NQO1 gene rs1800566 polymorphism (C-allele vs. T-allele model). There were significantly decreased association between NQO1 polymorphism and HCC susceptibility. The squares and horizontal lines correspond to the study-specific OR and 95% CI. The area of the squares reflects the weight (inverse of the variance). The diamond represents the summary OR and 95% CI.

**Figure 4:**
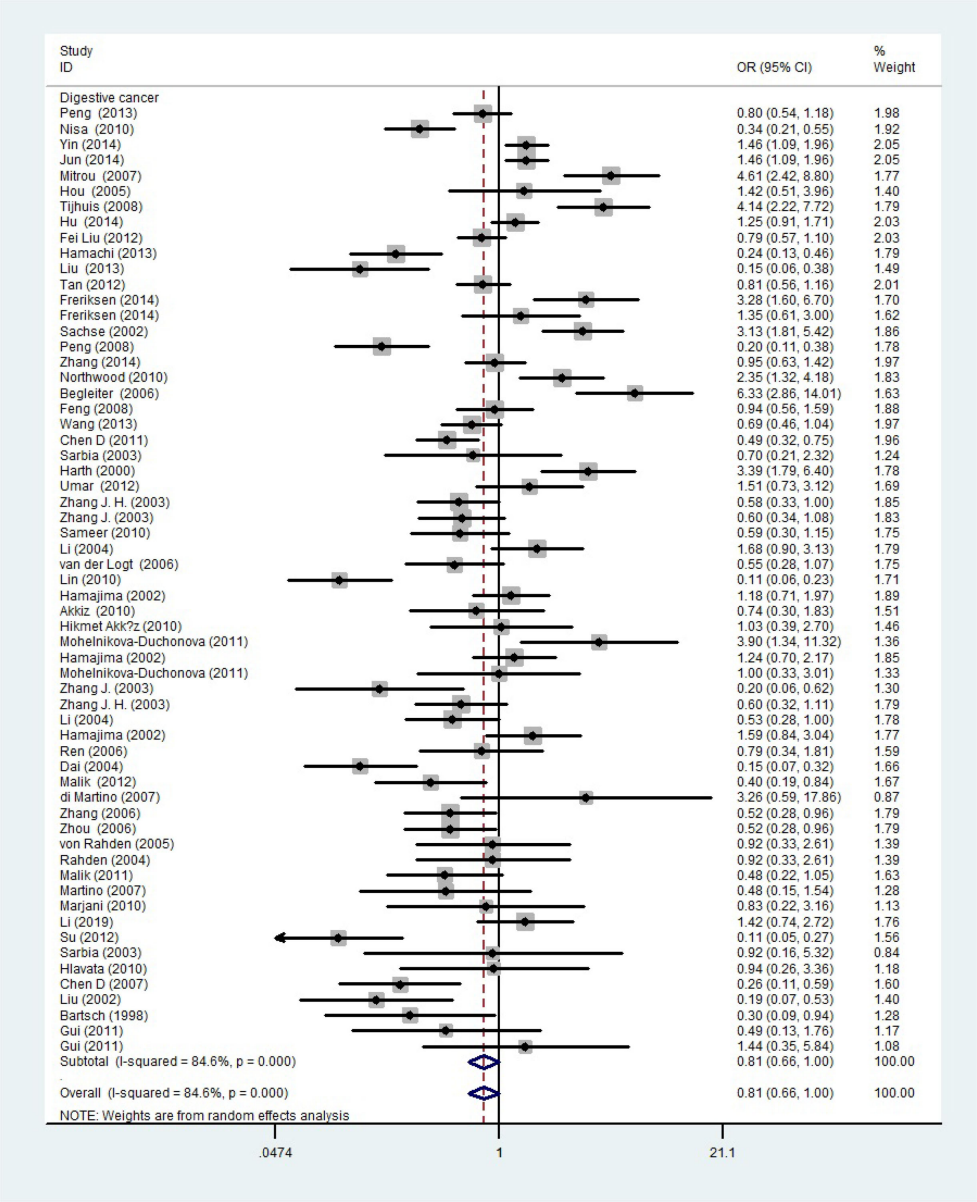
A forest plot showing the relationship between the NQO1 gene’s rs1800566 polymorphism and digestive cancer. There were significantly decreased association between NQO1 polymorphism and digestive cancer. The squares and horizontal lines correspond to the study-specific OR and 95% CI. The area of the squares reflects the weight (inverse of the variance). The diamond represents the summary OR and 95% CI.

**Figure 5:**
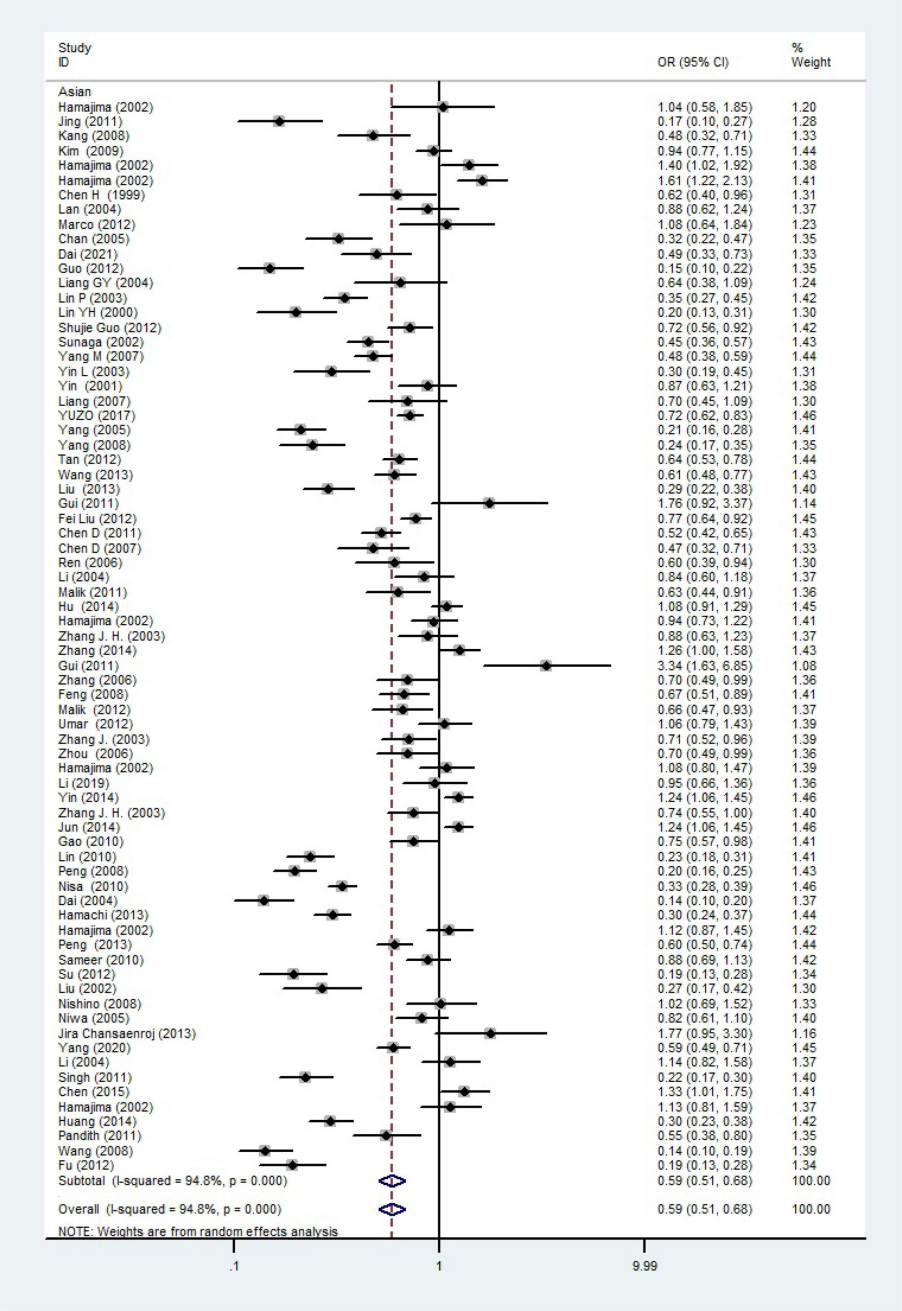
The relationship between cancer risk and rs1800566 polymorphism in the NQO1 gene in Asian population (C-allele vs. T-allele model). There were significantly decreased association between NQO1 polymorphism in Asian population. The squares and horizontal lines correspond to the study-specific OR and 95% CI. The area of the squares reflects the weight (inverse of the variance). The diamond represents the summary OR and 95% CI.

**Figure 6:**
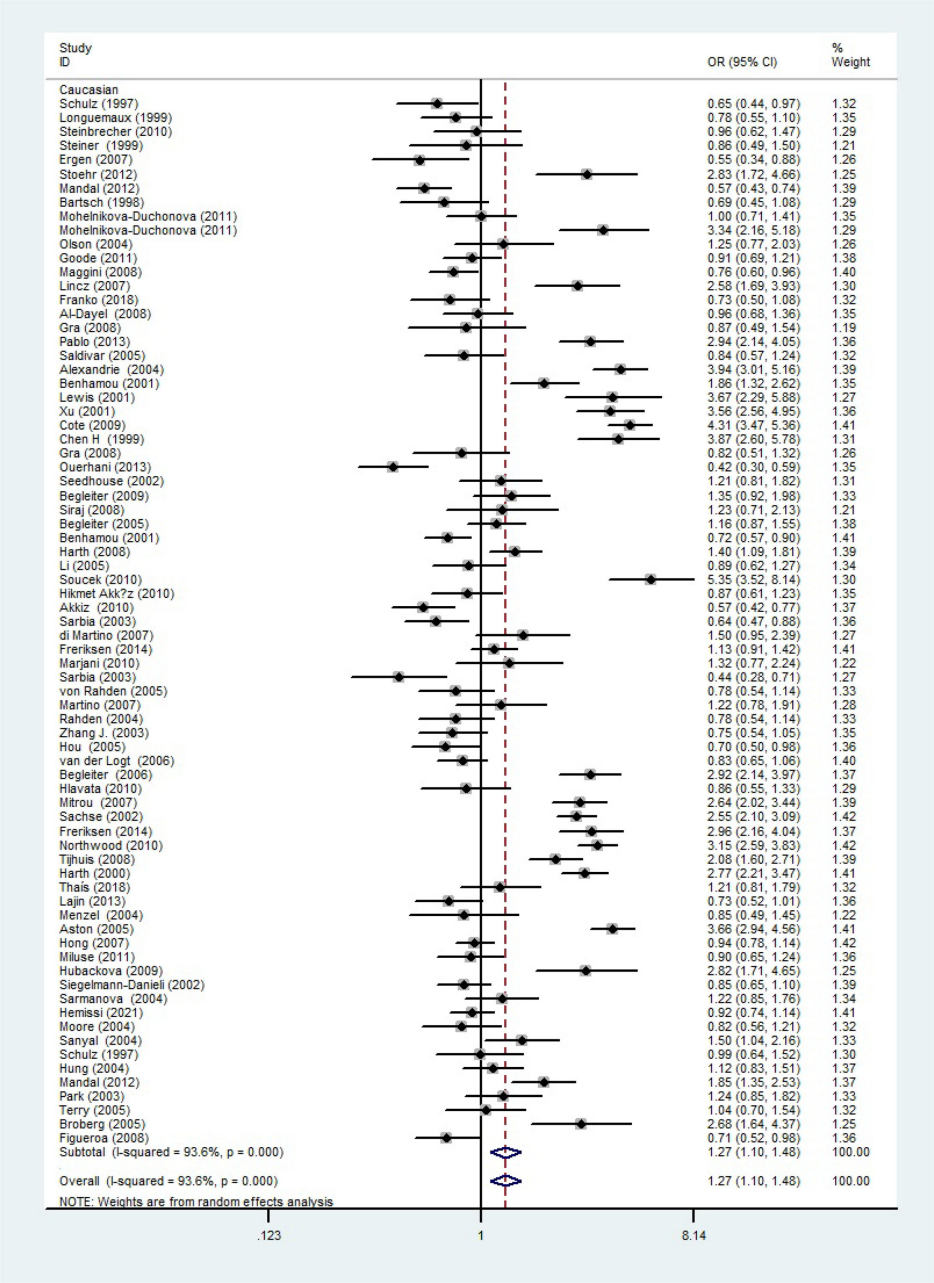
The relationship between cancer risk and the rs1800566 polymorphism in the NQO1 gene in Caucasians (C-allele vs. T-allele model). There were significantly increased association between NQO1 polymorphism in Caucasian population. The squares and horizontal lines correspond to the study-specific OR and 95% CI. The area of the squares reflects the weight (inverse of the variance). The diamond represents the summary OR and 95% CI.

**Figure 7:**
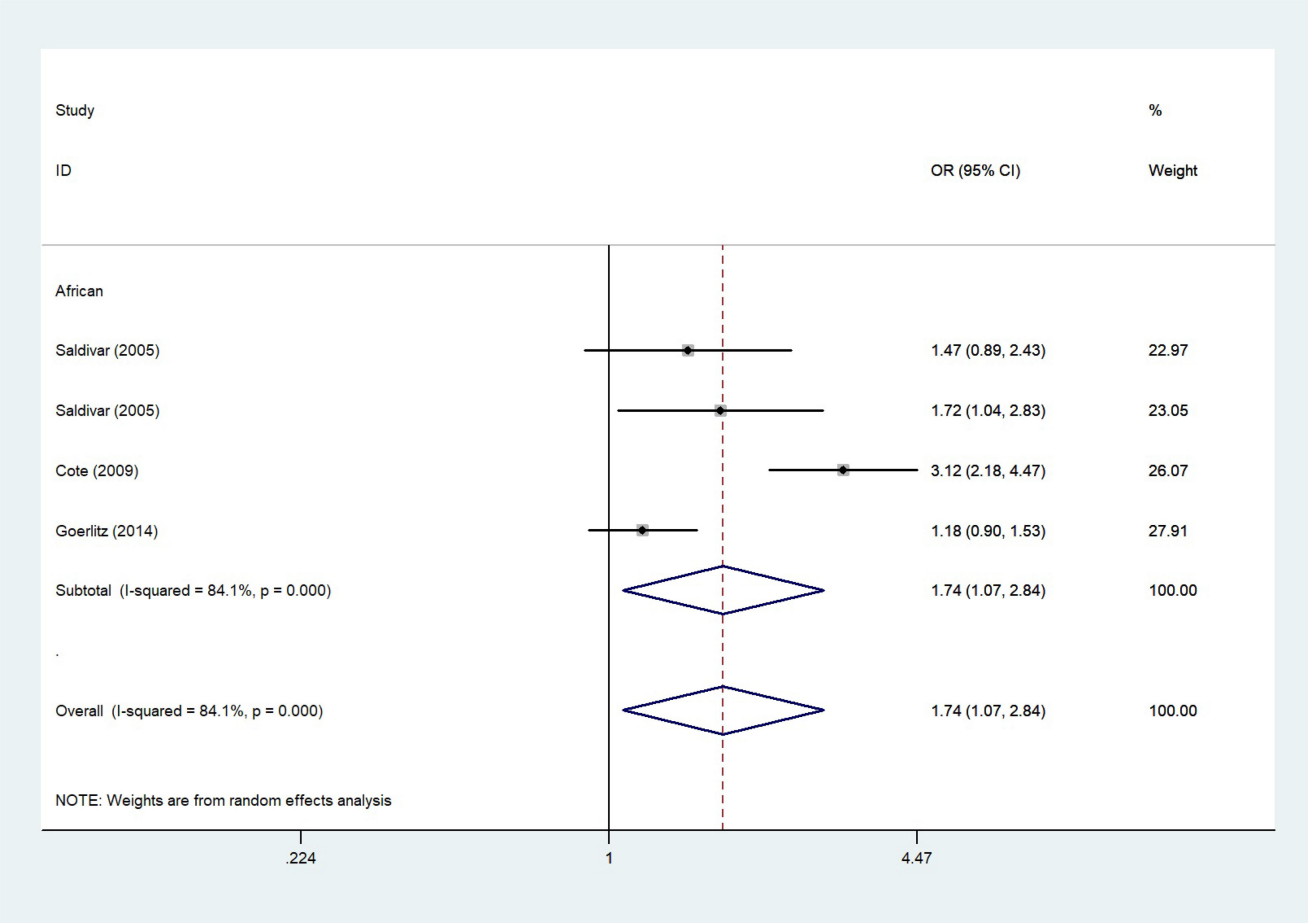
The relationship between cancer risk and the NQO1 gene rs1800566 polymorphism in Africans (C-allele vs. T-allele model). There were significantly increased association between NQO1 polymorphism in African individuals. The squares and horizontal lines correspond to the study-specific OR and 95% CI. The area of the squares reflects the weight (inverse of the variance). The diamond represents the summary OR and 95% CI.

**Figure 8:**
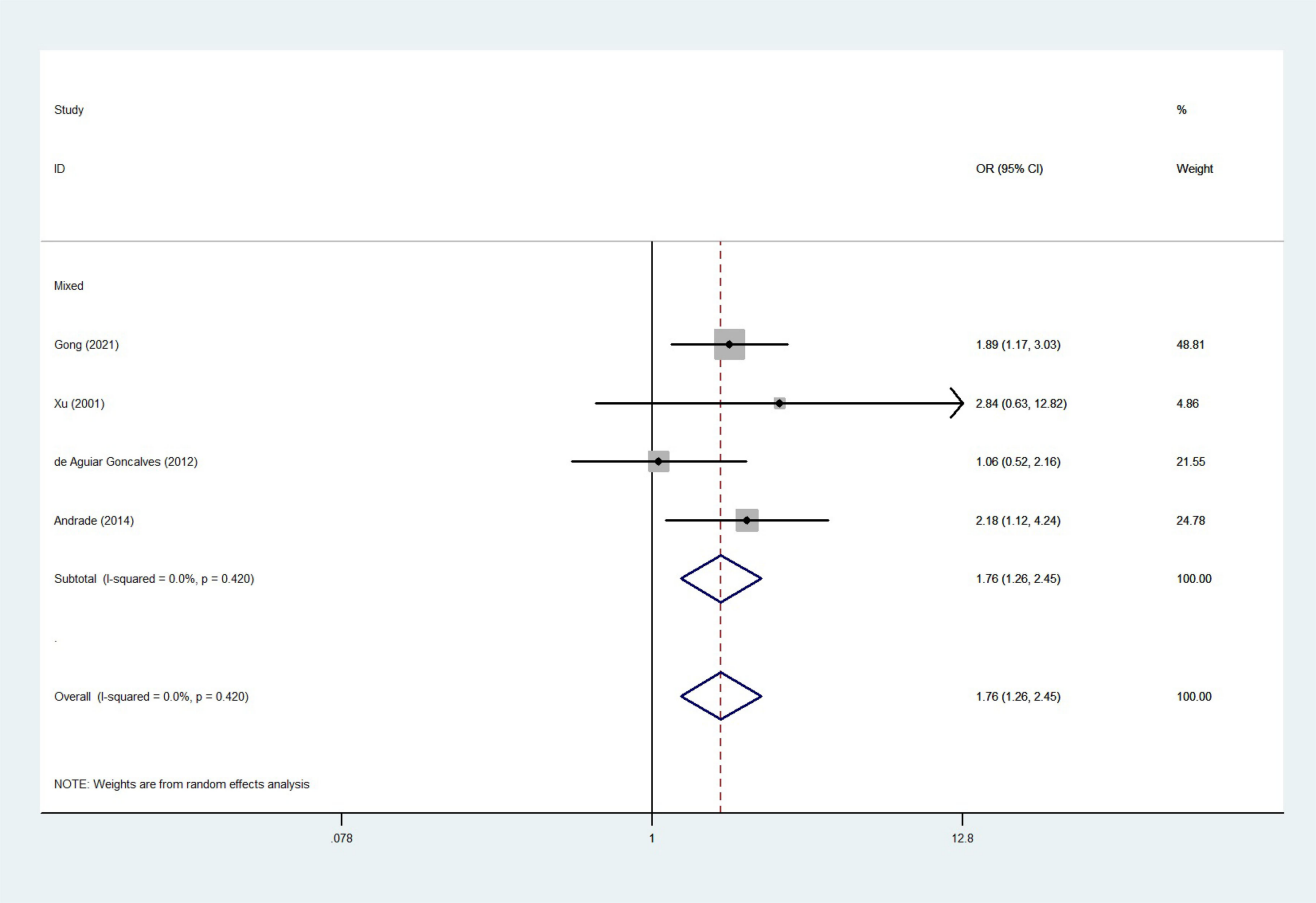
The relationship between cancer risk and the NQO1 gene rs1800566 polymorphism in mixed-race populations (CT vs. TT). There were significantly increased association between NQO1 polymorphism in Mixed population. The squares and horizontal lines correspond to the study-specific OR and 95% CI. The area of the squares reflects the weight (inverse of the variance). The diamond represents the summary OR and 95% CI.

**Figure 9:**
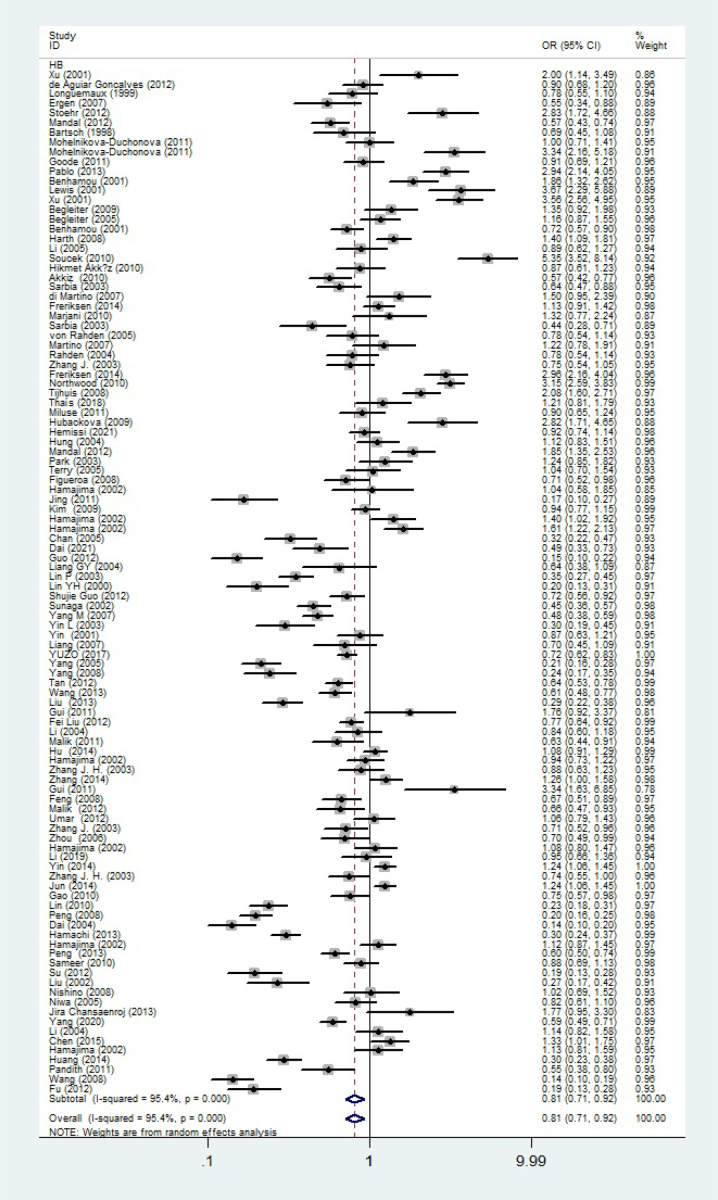
Forest plot about association between NQO1 gene rs1800566 polymorphism in HB subgroup (C-allele vs. T-allele model). There were significantly decreased association between NQO1 polymorphism in HB subgroup. The squares and horizontal lines correspond to the study-specific OR and 95% CI. The area of the squares reflects the weight (inverse of the variance). The diamond represents the summary OR and 95% CI.

The total risks of 43736 cancer cases and 56173 controls of NQO1 rs1800566 polymorphism are summarized in **Table 1**. Overall data showed that there was no significant association between NQO1 rs1800566 polymorphism and cancer susceptibility in all genetic models. However, significantly decreased associations were detected in cancer type subgroup (renal cell carcinoma: C-allele vs. T-allele, OR = 0.72, 95%CI = 0.56-0.94, *P*_heterogeneity_ = 0.491, *P* = 0.014; hepatocellular carcinoma: C-allele vs. T-allele, OR = 0.65, 95%CI = 0.49-0.87, *P*_heterogeneity_ < 0.001, *P* = 0.003, **Figure 3**; gastric cancer: C-allele vs. T-allele, OR = 0.77, 95%CI = 0.61-0.95, *P*_heterogeneity_ < 0.001, *P* = 0.017). When the studies were split into six systematic groups, a significantly decreased association between this polymorphism and digestive cancer was also detected (CT vs. TT, OR = 0.81, 95%CI = 0.66-1.00, *P*_heterogeneity_ *<*0.001, *P* = 0.049, **Figure 4**). A decreased risk was found for Asians in the ethnic subgroup (C-allele vs. T-allele, OR = 0.59, 95%CI = 0.51-0.68, *P*_heterogeneity_ < 0.001, *P* < 0.001, **Figure 5**). On the other hand, we observed significant associations in Caucasian population (C-allele vs. T-allele, OR = 1.27, 95%CI = 1.10-1.48, *P*_heterogeneity_ < 0.001, *P* = 0.001, **Figure 6**), Africans (C-allele vs. T-allele, OR = 1.74, 95%CI = 1.07-2.84, *P*_heterogeneity_ < 0.001, *P* = 0.026, **Figure 7**) and Mixed-race population (CT vs. TT, OR = 1.76, 95%CI = 1.26-2.45, *P*_heterogeneity_ = 0.420, *P* = 0.001, **Figure 8**). In addition, we assessed the OR for the NQO1 rs1800566 polymorphism based on stratification by source of control, and found decreased relationship in HB (C-allele vs. T-allele, OR = 0.81, 95%CI = 0.71-0.92, *P*_heterogeneity_ < 0.001, *P* = 0.001, **Figure 9**).

**Table 1:** Stratified analysis of NQO1 rs1800566 C/T variation on cancer susceptibility.

Variables	N	Case/	C-allele vs. T-allele	CT vs. TT	CC vs. TT	CC+CT vs. TT	CC vs. CT+TT
rs1800566 C/T		Control	OR (95% CI) *P*_h_ *P*	OR (95% CI) *P*_h_ *P*	OR (95% CI) *P*_h_ *P*	OR (95% CI) *P*_h_ *P*	OR (95% CI) *P*_h_ *P*
Total	176	43736/56173	0.90 (0.80-1.01) <0.001 0.076	0.89 (0.78-1.02)<0.001 0.095	0.81 (0.65-1.02) <0.001 0.069	0.86 (0.72-1.02) <0.001 0.089	0.87 (0.76-1.00) <0.001 0.055
HWE	156	39417/50405	0.90 (0.90-1.01) <0.001 0.076	0.89 (0.78-1.02)<0.001 0.095	0.81 (0.65-1.02) <0.001 0.069	0.86 (0.72-1.02) <0.001 0.089	0.87 (0.76-1.00) <0.001 0.055
Cancer Type (1)							
renal cell carcinoma	2	304/470	0.72 (0.56-0.94)0.491 0.014	0.74 (0.32-1.68)0.688 0.046	0.55 (0.25-1.24)0.955 0.149	0.61 (0.27-1.34) 0.883 0.215	0.69 (0.51-0.94) 0.387 0.018
prostate cancer	8	1211/2319	0.88 (0.44-1.77) <0.001 0.719	0.90 (0.50-1.63)0.003 0.728	0.74 (0.20-2.81) <0.001 0.658	0.83 (0.30-2.31) <0.001 0.723	0.83 (0.39-1.80) <0.001 0.643
pancreatic cancer	3	581/559	1.32 (0.55-3.17) <0.001 0.535	1.06 (0.25-4.57)0.005 0.935	1.49 (0.18-12.73) <0.001 0.714	1.30 (0.20-8.57) <0.001 0.784	1.41 (0.60-3.32) <0.001 0.428
ovarian cancer	2	1051/258	1.00 (0.76-1.32) 0.28 0.998	1.23 (0.61-2.48)0.717 0.563	1.20 (0.61-2.35)0.810 0.599	1.21 (0.62-2.36) 0.968 0.575	0.98 (0.67-1.44) 0.227 0.924
multiple myeloma	3	459/1279	0.97 (0.42-2.27) <0.001 0.944	1.25 (0.50-3.17)0.064 0.632	1.15 (0.22-6.01) <0.001 0.865	1.20 (0.35-4.10) 0.006 0.775	0.90 (0.29-2.87) <0.001 0.863
lymphoma	5	1260/1067	1.28 (0.82-2.01) <0.001 0.284	1.02 (0.53-1.99)0.003 0.948	1.47 (0.56-3.83) <0.001 0.433	1.24 (0.57-2.69) <0.001 0.587	1.43 (0.85-2.39) <0.001 0.177
lung cancer	40	9553/14270	0.97 (0.68-1.37) <0.001 0.862	1.19 (0.83-1.71) <0.001 0.353	1.11 (0.60-2.05) <0.001 0.751	1.07 (0.67-1.71) <0.001 0.774	0.90 (0.58-1.40) <0.001 0.637
leukemia	12	2084/2883	0.67 (0.37-1.21) <0.001 0.187	0.60 (0.26-1.36) <0.001 0.221	0.41 (0.12-1.47) <0.001 0.171	0.71 (0.22-2.27) <0.001 0.560	0.60 (0.35-1.04) <0.001 0.067
head and neck cancer	7	1904/2960	1.36 (0.83-2.25) <0.001 0.226	1.08 (0.67-1.73)0.212 0.767	1.64 (0.58-4.58) <0.001 0.349	1.43 (0.62-3.30) <0.001 0.400	1.42 (0.81-2.49) <0.001 0.223
hepatocellular carcinoma	7	2002/1942	0.65 (0.49-0.87) <0.001 0.003	0.66 (0.48-0.92)0.050 0.012	0.45 (0.26-0.77) <0.001 0.003	0.56 (0.38-0.83) 0.001 0.004	0.59 (0.42-0.84) <0.001 0.003
gastric cancer	10	2083/2829	0.77 (0.61-0.95) <0.001 0.017	0.67 (0.51-0.95)0.001 0.024	0.60 (0.39-0.92) <0.001 0.019	0.64 (0.45-0.92) <0.001 0.015	0.78 (0.60-1.02) <0.001 0.064
esophageal cancer	19	3981/5085	0.90 (0.78-1.03) <0.001 0.108	0.88 (0.68-1.15) <0.001 0.352	0.78 (0.58-1.06) <0.001 0.115	0.84 (0.64-1.10) <0.001 0.197	0.88 (0.76-1.04) <0.001 0.126
colorectal cancer	20	7868/9358	0.80 (0.50-1.27) <0.001 0.346	0.84 (0.48-1.47) <0.001 0.540	0.65 (0.25-1.71) <0.001 0.385	1.11 (0.93-1.32) <0.001 0.446	0.75 (0.45-1.27) <0.001 0.290
crvical cancer	6	989/1390	0.95 (0.67-1.36) <0.001 0.788	0.81 (0.50-1.31)0.063 0.395	0.91 (0.45-1.81) <0.001 0.777	0.88 (0.49-1.59) 0.002 0.681	0.97 (0.70-1.35) 0.017 0.858
breast cancer	12	3270/4392	1.14 (0.82-1.58) <0.001 0.451	1.68 (0.90-3.13) <0.001 0.103	1.40 (0.72-2.72) <0.001 0.326	1.02 (0.49-2.14) <0.001 0.951	1.09 (0.63-1.89) <0.001 0.751
bladder cancer	16	4566/4546	1.06 (0.67-1.67) <0.001 0.817	0.99 (0.56-1.76) <0.001 0.976	1.09 (0.44-2.71) <0.001 0.854	0.66 (0.36-1.21) <0.001 0.178	0.78 (0.51-1.22) <0.001 0.276
Cancer Type (2)							
Tumor of urinary system	26	6081/7335	0.81 (0.58-1.12) <0.001 0.204	1.19 (0.83-1.71) <0.001 0.125	0.64 (0.34-1.19) <0.001 0.158	0.71 (0.44-1.14) <0.001 0.157	0.79 (0.56-1.13) <0.001 0.200
Digestive cancer	61	16714/19929	0.84 (0.70-1.01) <0.001 0.067	0.81 (0.66-1.00) <0.001 0.049	0.70 (0.50-0.99) <0.001 0.042	0.76 (0.58-1.00) <0.001 0.047	0.82 (0.66-1.01) <0.001 0.066
Gynecological tumor	21	5223/6249	1.02 (0.76-1.36) <0.001 0.906	0.99 (0.70-1.40) <0.001 0.949	1.02 (0.59-1.78) <0.001 0.932	1.01 (0.65-1.57) <0.001 0.956	1.03 (0.73-1.44) <0.001 0.880
Hematological tumors	20	3803/5229	0.88 (0.61-1.26) <0.001 0.478	0.82 (0.52-1.29) <0.001 0.382	0.74 (0.36-1.51) <0.001 0.406	0.91 (0.49-1.70) <0.001 0.778	0.82 (0.55-1.20) <0.001 0.303
Respiratory tumors	40	9553/14270	0.97 (0.68-1.37) <0.001 0.8621	0.78 (0.57-1.07) <0.001 0.353	1.11 (0.60-2.05) <0.001 0.751	1.07 (0.67-1.72) <0.001 0.774	0.90 (0.58-1.40) <0.001 0.637
Head-neck tumors	7	1904/2960	0.90 (0.80-1.01) <0.001 0.226	1.08 (0.67-1.73)0.212 0.767	1.64 (0.58-4.58) <0.001 0.349	1.43 (0.62-3.30) <0.001 0.400	1.42 (0.81-2.49) <0.001 0.223
Ethnicity							
Asian	82	18372/26412	0.59 (0.51-0.68) <0.001 <0.001	0.60 (0.51-0.71) <0.001 <0.001	0.36 (0.27-0.48) <0.001 <0.001	0.48 (0.39-0.59) <0.001 <0.001	0.53 (0.45-0.63) <0.001 <0.001
African	6	1227/2037	1.74 (1.07-2.84) <0.001 0.026	1.88 (1.20-2.93)0.685 0.005	3.07 (1.36-6.91)0.032 0.007	1.11 (0.41-3.00) <0.001 0.843	1.85 (1.01-3.41) <0.001 0.047
Caucasian	83	22868/25893	1.27 (1.10-1.48) <0.001 0.001	1.23 (1.00-1.50) <0.001 0.046	1.46 (1.07-1.99) <0.001 0.018	1.54 (1.15-2.06) <0.001 0.004	1.32 (1.12-1.56) <0.001 0.001
Mixed	5	1269/1831	1.69 (0.99-2.89) <0.001 0.056	1.76 (1.26-2.45)0.420 0.001	2.82 (1.16-6.88) <0.001 0.022	1.39 (0.42-4.68) <0.001 0.592	1.77 (0.91-3.44) <0.001 0.091
Source of control							
HB	115	28878/36564	0.81 (0.71-0.92) <0.001 0.001	0.78 (0.68-0.90) <0.001 0.001	0.67 (0.52-0.85) <0.001 0.001	0.71 (0.59-0.86) <0.001 <0.001	0.77 (0.66-0.90) <0.001 0.001
PB	61	14858/19609	1.13 (0.90-1.42) <0.001 0.286	1.23 (0.93-1.63) <0.001 0.146	1.33 (0.85-2.09) <0.001 0.212	1.27 (0.87-1.86) <0.001 0.215	1.12 (0.87-1.45) <0.001 0.374

P_h_: value of Q-test for heterogeneity test; P: Z-test for the statistical significance of the OR.

### Publication sensitivity and bias analysis

**Figure 10:**
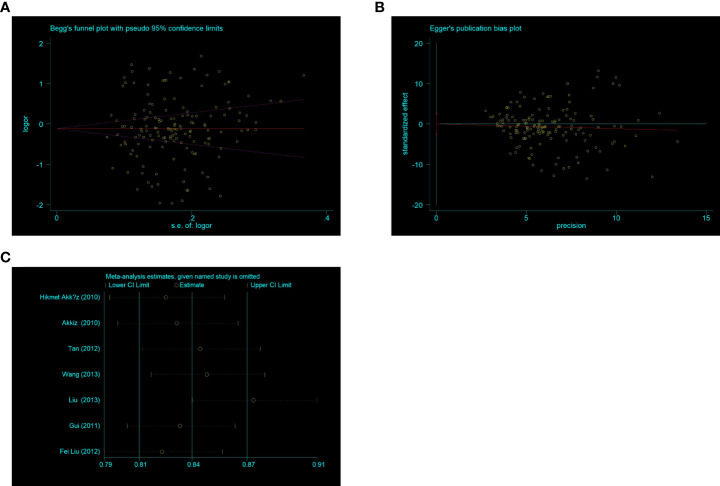
**(A)** Begg’s funnel plot for publication bias (C -allele vs T-allele). **(B)** The plot of Egger’s publishing bias (C -allele vs T-allele). **(C)** The sensitivity analysis between NQO1 rs1800566 polymorphism and HCC risk (C allele vs T-allele).

To assess publication bias, Begg’s and Egger’s tests were used. There was no indication about publication bias in both tests (such as C-allele vs. T-allele, t = 0.02, *P* = 0.986 for Egger’s test; and z = 0.78, *P* = 0.434 for Begg’s test, **Figures 10A, B** **)** (**Table 2**). To find out whether progressively deleting each individual study had an impact on the pooled OR, a sensitivity analysis was carried out. According to the results, no single study had a significant effect on the overall OR (**Figure 10C**).

**Table 2:** Publication bias tests (Begg’s funnel plot and Egger’s test for publication bias test).

Egger’s test						Begg’s test	
Genetic type	Coefficient	Standard error	*t*	*P*-value	95 %CI of intercept	*z*	*P*-value
C-allele vs. T-allele	0.023	1.252	0.02	0.986	(-2.450, 2.500)	0.78	0.434
CT vs. TT	-0.041	0.440	-0.09	0.927	(-0.910, 0.828)	0.77	0.443
CC vs. TT	-0.183	0.799	-0.23	0.819	(-1.761, 1.395)	0.66	0.509
CC+CT vs. TT	-0.043	0.622	-0.07	0.945	(-1.272, 1.186)	0.80	0.423
CC vs. CT+TT	-0.552	1.131	-0.49	0.626	(-2.786, 1.681)	0.06	0.950

for NQO1 rs1800566 polymorphism.

### Meta-regression

**Figure 11:**
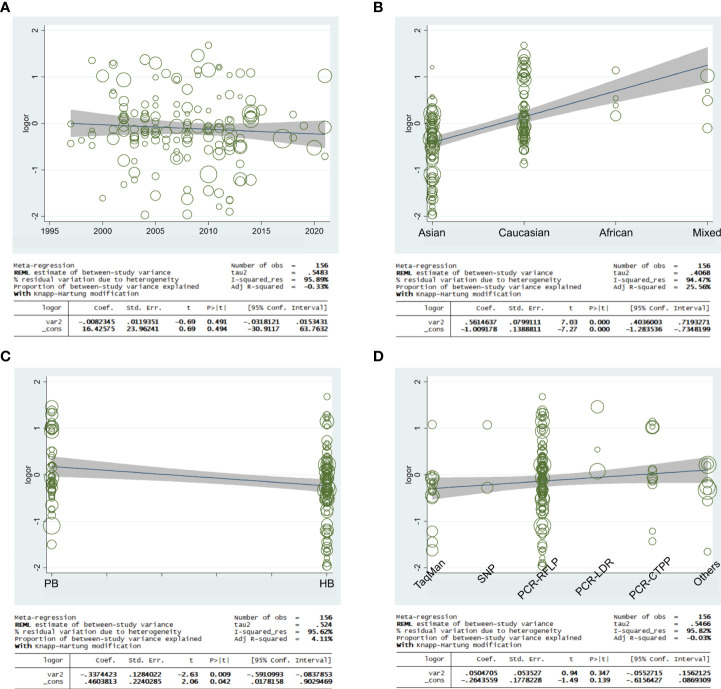
Random-effect meta-regression was used to examine the log odds ratio in relation to publication year **(A)**, regular ethnicity **(B)**, source of control **(C)**, and genotyping methodologies **(D)**.

Meta-regression analysis was applied to access if the heterogeneity was found in the current study. Based on the final analysis, the regression coefficients of allele models based on ethnicity and source of control (C allele vs. T allele) were both less than 0.05, which indicated that heterogeneity may be generated from ethnicity or source of control subgroup (**Figure 11**).

## Discussion

Numerous studies have confirmed that redox-regulated flavoenzyme NQO1 can monitor the redox status in cells and protect against oxidative stress and carcinogenesis by stabilizing p53. NQO1 was first discovered to be inducible by a variety of compounds, and many of which showed cancer-fighting properties (23). Many studies have shown that rs1800566 polymorphism in NQO1 gene was significantly linked with a variety of tumors, and many factors may influence, including origin, ethnicity, and environmental susceptibility. However, the results have been inconsistent or showed insignificant correlations partly due to low sample sizes or signal tumor type, although several meta-analyses have been reported. Our comprehensive study between cancer susceptibility and NQO1 rs1800566 polymorphism was tried best to conduct convince conclusions based on a larger sample size.

The interaction between genes and the environment is believed to play a vital role in the etiology of many types of cancer. Many components of known environmental risk factors, including environmental tobacco smoke and environmental air pollution, are metabolized into more carcinogenic or detoxifying products in the organism (24). NQO1 may have carcinogenic and mutagenic effects caused by quinone and its metabolic precursors (25). Benzene is an occupational blood toxin, NQO1 can convert benzene-derived quinones to less toxic hydroquinones (26). Zhang et al. also proved that NQO1 rs1800566 CC genotype can save 1,4-BQ (Benzoquinone) induced DNA damage through experiments (27), and this benzene metabolite is believed to be related to bone morrow toxicity and leukemia. Lung cancer is also associated with the metabolism of some carcinogens, such as Benzo(a)pyrene (BP), and the formation of BP quinone-DNA adduct is prevented by NQO1 (28). Cigarette smoke is the main source of airway oxidative stress. The disappearance of antioxidant/oxidant homeostasis leads to the development of lung inflammation and tumor. In addition, the homeostasis of NQO1 is also affected by cigarette smoke (29). Nevertheless, in above process, NQO1 not only provides detoxification, but also participates in the metabolic reduction of some carcinogens, for example, dicoumarol (an inhibitor of the NQO 1 activity) partially prevents the metabolic activation of some promutagens (30). The NQO1 rs1800566 polymorphism led to the replacement of proline to serine, thus resulted in a significant reduction in its enzyme activity (31), which may participate in above biological process. Besides, NQO1also acts as a stabilizer for tumor suppressors, which may play a role in carcinogenesis (32). Whether NQO1 exclusively serves as a protective enzyme or plays a role in generating reactive metabolites, it should be investigated through toxicity, mutagenicity for cancer development.

Beta-lapachone (an o-naphthoquinone) induces a novel caspase- or p53-independent apoptotic pathway dependent on NQO1, which can target high level enzymes in various tumors, thus showing great application prospects, however, there are also several drawbacks, such as toxicological effects in normal tissues (33). It is possible to develop a precursor drug that can only be converted into Beta-lapachone in the tumor microenvironment, but also increase the target of Beta-lapachone. The combination β-lap and other therapeutic strategies can also expand clinical application and improve the therapeutic effect (34–36). The role of Beta-lapachone depends on NQO1 activity. NQO1 activity and protein expression level can be obtained through tumor biopsy, but NQO1 protein expression is affected by many factors. Single biopsy result may not be accurate, which may influence the therapeutic effect (37). The effect of Beta-lapachone depends on the production of active oxygen species, which in NRF2/KEAP1 mutant cells will be actively eliminated, finally, resulting in drug resistance. Drug resistance needs to be eliminated by inhibiting the thioredoxin-dependent system or copper-zinc superoxide dismutase (38). The application of Beta-lapachone has inspired us in many research directions. So far, many drugs targeting NQO1 have been opened up, and their target and efficacy have been enhanced. The focus of our article about NQO1 rs180566 polymorphism may help for clinical diagnosis and treatment of tumors.

Subsequently, neither NQO1 rs1800566 polymorphisms nor any other NQO1 polymorphisms were found to be substantially linked with tumor risk in the genetic model. However, when we divided the subgroups, we noticed significantly decreased associations were found among renal cell carcinoma, hepatocellular carcinoma, gastric cancer and NQO1 rs1800566 polymorphism. Moreover, in the ethnicity subgroup, NQO1 rs1800566 polymorphism played as a protective role in Asians, while completely opposite correlations were observed in Caucasian, African, and mixed populations. When the subgroups were divided according to the source of the control group, the source of PB was correlated significantly with NQO1 rs1800566 polymorphism. This is not exactly the same as the results of Lajin et al. (17). In their study, on stratifying by cancer subgroup, they found significantly increased correlation between two type of cancer (bladder cancer and gastric cancer) and NQO1 polymorphism. But our study the significantly decreased associations were found in renal cell carcinoma, gastric cancer, and hepatocellular carcinoma. In the analysis of subgroups stratified by race, they found that there was a statistically significant correlation among Caucasian subgroups. The correlation in the TT vs. CC model was the strongest, and we obtained the same results. In addition, we also found that African and mixed populations also showed significant increased correlations for NQO1 rs1800566 polymorphism. More importantly, we found that the significantly decreased correlation in Asian subgroup. The possible reasons of those results may be as follows: in the beginning, there was heterogeneity in the included population. Because we found that Caucasians showed high heterogeneity in some allele frequencies, and different ethnicities had different genetic structures. Second, Different populations may be susceptible to rs1800566 polymorphism. Third, environmental factors may play a greater role between rs1800566 polymorphism and tumors. Many studies have been believed that environmental factors lead to the change of alleles, which may be explained the inactivation of the NQO1 enzyme may change the susceptibility to some types of cancer. Fourth, there were a few samples from Africa, mixed population, and some types of cancer, possibility of false-positive results may be existed. The digestive system cancer had a weaker correlation with NQO1 rs1800566 polymorphism, which were classified malignancies into six different systems. This was contrary to the results from Upendra et al. (39), in which they showed that rs1800566 polymorphism of NQO1 was a risk factor for digestive system tumors. The reasons maybe: a possible heterogeneity among different individuals was proved in the same population; and due to the study from Upendra et al. (39), which were not included African and mixed populations.

In current study, we focused on one of rs1800566 polymorphism in the NQO1 gene, but in fact there had other sites that may affect tumor susceptibility, such as rs1131341, which were reported to be associated with an increased risk for acute lymphoblastic leukemias (ALL) (40, 41). This SNP can result in alternative splice sites of mRNA, deletion of exon 4 and to create a protein lacking the quinone binding site, for which enzyme activity differs according to the substrate (42). Although no study has proved rs1131341 polymorphism played a role in tumor other than ALL, another variant of high linkage disequilibrium (LD) may be the cause of these changes, and the difference in LD between populations can also be explained for the high heterogeneity.

Our study had some limitations. First, the combined effect of environment and NQO1 gene should be paid attention, but the impact of these factors was not described in this study. Moreover, the carcinogenic effect of a single factor in a certain population may be masked by other stronger carcinogenic factors, and there may be synergistic or antagonistic effects between various factors. Therefore, future studies should concentrate on how interact between the environment and NQO1 rs1800566 polymorphism. Third, we should help to establish a unified research model or database to obtain a larger sample size, exclude the influence of other factors, categorize more reasonable ethnic subgroups to eliminate heterogeneity as much as possible, comprehensive analyze multiple factors, and change the plan for examination and treatment according to different populations.

## Conclusion

The present comprehensive meta-analysis suggested the NQO1 rs1800566 polymorphism was an important genetic factor in the risk of cancer, especially in Caucasians, moreover, decreased associations in gastric cancer, hepatocellular carcinoma and renal cell carcinoma were proved. More in-depth studies in the future should be confirmed between NQO1 rs1800566 polymorphism and cancer susceptibility.

## Data availability statement

The datasets presented in this study can be found in online repositories. The names of the repository/repositories and accession number(s) can be found in the article/**Supplementary Material**.

## Author contributions

HW and HZ were major contributors in writing the manuscript. HW created all the figures. HW and HZ performed the literature search. LZ and YM made substantial contributions to the design of the manuscript and revised it critically for important intellectual content. All authors contributed to the article and approved the submitted version.

## Funding

This work was supported by National Natural Science Foundation (No. 81802576), Wuxi City Medical Young Talent (No. QNRC043), Wuxi Commission of Health and Family Planning (No. T202102, T202024, J202012, Z202011), the Science and Technology Development Fund of Wuxi (No. N20202021), and Jiangnan University Wuxi School of Medicine (No. 1286010242190070) and Talent plan of Taihu Lake in Wuxi (Double Hundred Medical Youth Professionals Program) from Health Committee of Wuxi (No. BJ2020061). Clinical trial of Affiliated Hospital of Jiangnan University (No. LCYJ202227), Research topic of Jiangsu Health Commission (Z2022047).

## Conflict of interest

The authors declare that the research was conducted in the absence of any commercial or financial relationships that could be construed as a potential conflict of interest.

## Publisher’s note

All claims expressed in this article are solely those of the authors and do not necessarily represent those of their affiliated organizations, or those of the publisher, the editors and the reviewers. Any product that may be evaluated in this article, or claim that may be made by its manufacturer, is not guaranteed or endorsed by the publisher.

## Supplementary material

Supplementary Figure 1
**(A)** The MAF for the NQO1 rs1800566 polymorphism from the online 1000 Genome. **(B)** C- and T-allele frequencies in the case and control groups. **(C)** The genotype distribution from the GTEx Portal website (the site’s main page is located at https://www.gtexportal.org). **(D)** The risk frequency of rs1800566 polymorphism in many types of diseases from TCGA database.

## References

[1] Sung H Ferlay J Siegel RL Laversanne M Soerjomataram I Jemal A. Global cancer statistics 2020: GLOBOCAN estimates of incidence and mortality worldwide for 36 cancers in 185 countries. CA Cancer J Clin (2021) 71:209–49. doi: 10.3322/caac.21660 33538338

[2] Bray F Laversanne M Weiderpass E Soerjomataram I. The ever-increasing importance of cancer as a leading cause of premature death worldwide. Cancer (2021) 127:3029–30. doi: 10.1002/cncr.33587 34086348

[3] Risch NJ. Searching for genetic determinants in the new millennium. Nature (2000) 405:847–56. doi: 10.1038/35015718 10866211

[4] Botstein D Risch N. Discovering genotypes underlying human phenotypes: past successes for mendelian disease, future approaches for complex disease. Nat Genet (2003) 33 Suppl:228–37. doi: 10.1038/ng1090 12610532

[5] Erichsen HC Chanock SJ. SNPs in cancer research and treatment. Br J Cancer (2004) 90:747–51. doi: 10.1038/sj.bjc.6601574 PMC2410167 14970847

[6] Zhu Y Spitz MR Amos CI Lin J Schabath MB Wu X. An evolutionary perspective on single-nucleotide polymorphism screening in molecular cancer epidemiology. Cancer Res (2004) 64:2251–7. doi: 10.1158/0008-5472.CAN-03-2800 15026370

[7] Sud A Kinnersley B Houlston RS. Genome-wide association studies of cancer: current insights and future perspectives. Nat Rev Cancer (2017) 17:692–704. doi: 10.1038/nrc.2017.82 29026206

[8] Zugazagoitia J Guedes C Ponce S Ferrer I Molina-Pinelo S Paz-Ares L. Current challenges in cancer treatment. Clin Ther (2016) 38:1551–66. doi: 10.1016/j.clinthera.2016.03.026 27158009

[9] Dinkova-Kostova AT Talalay P. NAD(P)H:quinone acceptor oxidoreductase 1 (NQO1), a multifunctional antioxidant enzyme and exceptionally versatile cytoprotector. Arch Biochem Biophys (2010) 501:116–23. doi: 10.1016/j.abb.2010.03.019 PMC2930038 20361926

[10] Zhang K Chen D Ma K Wu X Hao H Jiang S. NAD(P)H:Quinone oxidoreductase 1 (NQO1) as a therapeutic and diagnostic target in cancer. J Med Chem (2018) 61:6983–7003. doi: 10.1021/acs.jmedchem.8b00124 29712428

[11] Asher G Tsvetkov P Kahana C Shaul Y. A mechanism of ubiquitin-independent proteasomal degradation of the tumor suppressors p53 and p73. Genes Dev (2005) 19:316–21. doi: 10.1101/gad.319905 PMC546509 15687255

[12] Moscovitz O Tsvetkov P Hazan N Michaelevski I Keisar H Ben-Nissan G. A mutually inhibitory feedback loop between the 20S proteasome and its regulator, NQO1. Mol Cell (2012) 47:76–86. doi: 10.1016/j.molcel.2012.05.049 22793692

[13] Tian L Xiao P Zhou B Chen Y Kang L Wang Q. Influence of NQO1 polymorphisms on warfarin maintenance dose: A systematic review and meta-analysis (rs1800566 and rs10517). Cardiovasc Ther (2021) 2021:5534946. doi: 10.1155/2021/5534946 34457036 PMC8376459

[14] Dong D Zou Y Zhang P Wu Z. Systematic analyses and comprehensive field synopsis of genetic association studies in hepatocellular carcinoma. Oncotarget (2016) 7:45757–63. doi: 10.18632/oncotarget.9937 PMC5216758 27304192

[15] Guo ZJ Feng CL. The NQO1 rs1800566 polymorphism and risk of bladder cancer: evidence from 6,169 subjects. Asian Pac J Cancer Prev (2012) 13:6343–8. doi: 10.7314/APJCP.2012.13.12.6343 23464456

[16] Ding R Lin S Chen D. Association of NQO1 rs1800566 polymorphism and the risk of colorectal cancer: A meta-analysis. Int J Colorectal Dis (2012) 27:885–92. doi: 10.1007/s00384-011-1396-0 22215148

[17] Lajin B Alachkar A. The NQO1 polymorphism C609T (Pro187Ser) and cancer susceptibility: A comprehensive meta-analysis. Br J Cancer (2013) 109:1325–37. doi: 10.1038/bjc.2013.357 PMC3778271 23860519

[18] Higgins JP Thompson SG. Quantifying heterogeneity in a meta-analysis. Stat Med (2002) 21:1539–58. doi: 10.1002/sim.1186 12111919

[19] Mantel N Haenszel W. Statistical aspects of the analysis of data from retrospective studies of disease. J Natl Cancer Inst (1959) 22:719–48. 13655060

[20] DerSimonian R Laird N. Meta-analysis in clinical trials. Control Clin Trials (1986) 7:177–88. doi: 10.1016/0197-2456(86)90046-2 3802833

[21] Hayashino Y Noguchi Y Fukui T. Systematic evaluation and comparison of statistical tests for publication bias. J Epidemiol (2005) 15:235–43. doi: 10.2188/jea.15.235 PMC7904376 16276033

[22] Musso G Sircana A Saba F Cassader M Gambino R. Assessing the risk of ketoacidosis due to sodium-glucose cotransporter (SGLT)-2 inhibitors in patients with type 1 diabetes: A meta-analysis and meta-regression. PloS Med (2020) 17:e1003461. doi: 10.1371/journal.pmed.1003461 33373368 PMC7771708

[23] Ross D Siegel D. The diverse functionality of NQO1 and its roles in redox control. Redox Biol (2021) 41:101950. doi: 10.1016/j.redox.2021.101950 33774477 PMC8027776

[24] Vineis P Veglia F Garte S Malaveille C Matullo G Dunning A. Genetic susceptibility according to three metabolic pathways in cancers of the lung and bladder and in myeloid leukemias in nonsmokers. Ann Oncol (2007) 18:1230–42. doi: 10.1093/annonc/mdm109 17496311

[25] Prochaska HJ Talalay P Sies H. Direct protective effect of NAD(P)H:quinone reductase against menadione-induced chemiluminescence of postmitochondrial fractions of mouse liver. J Biol Chem (1987) 262:1931–4. doi: 10.1016/S0021-9258(18)61597-2 2434474

[26] Larson RA Wang Y Banerjee M Wiemels J Hartford C Le Beau MM. Prevalence of the inactivating 609C–>T polymorphism in the NAD(P)H:quinone oxidoreductase (NQO1) gene in patients with primary and therapy-related myeloid leukemia. Blood (1999) 94:803–7. doi: 10.1182/blood.V94.2.803 10397748

[27] Zhang J Yin L Liang G Liu R Pu Y. Detection of quinone oxidoreductase 1 (NQO1) single-nucleotide polymorphisms (SNP) related to benzene metabolism in immortalized b lymphocytes from a Chinese han population. J Toxicol Environ Health A (2010) 73:490–8. doi: 10.1080/15287390903523436 20391128

[28] Joseph P Jaiswal AK. NAD(P)H:quinone oxidoreductase1 (DT diaphorase) specifically prevents the formation of benzo[a]pyrene quinone-DNA adducts generated by cytochrome P4501A1 and P450 reductase. Proc Natl Acad Sci U.S.A. (1994) 91:8413–7. doi: 10.1073/pnas.91.18.8413 PMC44616 8078896

[29] Stringer KA Freed BM Dunn JS Sayers S Gustafson DL Flores SC. Particulate phase cigarette smoke increases MnSOD, NQO1, and CINC-1 in rat lungs. Free Radic Biol Med (2004) 37:1527–33. doi: 10.1016/j.freeradbiomed.2004.08.008 15477004

[30] De Flora S Bennicelli C Camoirano A Serra D Hochstein P. Influence of DT diaphorase on the mutagenicity of organic and inorganic compounds. Carcinogenesis (1988) 9:611–7. doi: 10.1093/carcin/9.4.611 2451576

[31] Lienhart WD Gudipati V Uhl MK Binter A Pulido SA Saf R. Collapse of the native structure caused by a single amino acid exchange in human NAD(P)H:quinone oxidoreductase(1). FEBS J (2014) 281:4691–704. doi: 10.1111/febs.12975 PMC4612375 25143260

[32] Asher G Lotem J Kama R Sachs L Shaul Y. NQO1 stabilizes p53 through a distinct pathway. Proc Natl Acad Sci U.S.A. (2002) 99:3099–104. doi: 10.1073/pnas.052706799 PMC122479 11867746

[33] Reinicke KE Bey EA Bentle MS Pink JJ Ingalls ST Hoppel CL. Development of beta-lapachone prodrugs for therapy against human cancer cells with elevated NAD(P)H:quinone oxidoreductase 1 levels. Clin Cancer Res (2005) 11:3055–64. doi: 10.1158/1078-0432.CCR-04-2185 15837761

[34] Singh N Pay SL Bhandare SB Arimpur U Motea EA. Therapeutic strategies and biomarkers to modulate PARP activity for targeted cancer therapy. Cancers (Basel) (2020) 12 :972. doi: 10.3390/cancers12040972 PMC7226473 32295316

[35] Choi EK Terai K Ji IM Kook YH Park KH Oh ET. Upregulation of NAD(P)H:quinone oxidoreductase by radiation potentiates the effect of bioreductive beta-lapachone on cancer cells. Neoplasia (2007) 9:634–42. doi: 10.1593/neo.07397 PMC1950433 17786182

[36] Bentle MS Reinicke KE Dong Y Bey EA Boothman DA. Nonhomologous end joining is essential for cellular resistance to the novel antitumor agent, beta-lapachone. Cancer Res (2007) 67:6936–45. doi: 10.1158/0008-5472.CAN-07-0935 17638905

[37] Siegel D Yan C Ross D. NAD(P)H:quinone oxidoreductase 1 (NQO1) in the sensitivity and resistance to antitumor quinones. Biochem Pharmacol (2012) 83:1033–40. doi: 10.1016/j.bcp.2011.12.017 PMC3482497 22209713

[38] Torrente L Prieto-Farigua N Falzone A Elkins CM Boothman DA Haura EB. Inhibition of TXNRD or SOD1 overcomes NRF2-mediated resistance to β-lapachone. Redox Biol (2020) 30:101440. doi: 10.1016/j.redox.2020.101440 32007910 PMC6997906

[39] Yadav U Kumar P Rai V. NQO1 gene C609T polymorphism (dbSNP: rs1800566) and digestive tract cancer risk: A meta-analysis. Nutr Cancer (2018) 70:557–68. doi: 10.1080/01635581.2018.1460674 29652514

[40] Eguchi-Ishimae M Eguchi M Ishii E Knight D Sadakane Y Isoyama K. The association of a distinctive allele of NAD(P)H:quinone oxidoreductase with pediatric acute lymphoblastic leukemias with MLL fusion genes in Japan. Haematologica (2005) 90:1511–5. doi: 10.3324/%x 16266898

[41] Krajinovic M Sinnett H Richer C Labuda D Sinnett D. Role of NQO1, MPO and CYP2E1 genetic polymorphisms in the susceptibility to childhood acute lymphoblastic leukemia. Int J Cancer (2002) 97:230–6. doi: 10.1002/ijc.1589 11774269

[42] Guha N Chang JS Chokkalingam AP Wiemels JL Smith MT Buffler PA. NQO1 polymorphisms and *de novo* childhood leukemia: A HuGE review and meta-analysis. Am J Epidemiol (2008) 168:1221–32. doi: 10.1093/aje/kwn246 PMC2727266 18945694

