# Supplementary material for: Research on the effects of rs1800566 C/T polymorphism of NAD(P)H quinone oxidoreductase 1 gene on cancer risk involves analysis of 43,736 cancer cases and 56,173 controls
Source: Front Oncol. 2022 Oct 19;12:980897. doi: 10.3389/fonc.2022.980897 (PMC9627178; doi:10.3389/fonc.2022.980897)
Supplement: Supplementary file 1 [file Table_1.docx]

**Supplementary Table 1** Characteristics of studies of *NQO1* gene rs1800566 C/T polymorphism and cancer risk included in our meta-analysis

| First author | | Year | Origin | Cancer type (1) | Cancer type (2) | Ethnicity | Source | Case | Control | HWE | Method |
| --- | --- | --- | --- | --- | --- | --- | --- | --- | --- | --- | --- |
| Longuemaux[1] | 1999 | | France | renal cell carcinoma | Tumor of urinary system | Caucasian | HB | 173 | 210 | 0.998 | PCR-RFLP |
| Schulz[2] | 1997 | | Germany | renal cell carcinoma | Tumor of urinary system | Caucasian | PB | 131 | 260 | 0.76 | PCR-RFLP |
| Ergen[3] | 2007 | | Turkey | prostate cancer | Tumor of urinary system | Caucasian | HB | 45 | 513 | 0.893 | PCR-RFLP |
| Gong[4] | 2021 | | USA | prostate cancer | Tumor of urinary system | Mixed | PB | 449 | 640 | 0.08 | PCR-CTPP |
| Hamajima[5] | 2002 | | Japan | prostate cancer | Tumor of urinary system | Asian | HB | 56 | 40 | 0.673 | Taqman |
| Jing[6] | 2011 | | China | prostate cancer | Tumor of urinary system | Asian | NS | 45 | 250 | 0.113 | PCR-RFLP |
| Mandal[7] | 2012 | | India | prostate cancer | Tumor of urinary system | Caucasian | HB | 195 | 492 | 0.011 | NA |
| Steinbrecher[8] | 2010 | | Germany | prostate cancer | Tumor of urinary system | Caucasian | PB | 248 | 100 | 0.46 | PCR-RFLP |
| Steiner[9] | 1999 | | Germany | prostate cancer | Tumor of urinary system | Caucasian | PB | 54 | 232 | 0.84 | PCR-RFLP |
| Stoehr[10] | 2012 | | Germany | prostate cancer | Tumor of urinary system | Caucasian | HB | 119 | 52 | 0.27 | PCR-RFLP |
| Broberg[11] | 2005 | | Sweden | bladder cancer | Tumor of urinary system | Caucasian | PB | 61 | 241 | 0.5 | PCR |
| Figueroa[12] | 2008 | | Spain | bladder cancer | Tumor of urinary system | Caucasian | HB | 1128 | 156 | 0.44 | MALDI-TOF |
| Goerlitz[13] | 2014 | | Egypt | bladder cancer | Tumor of urinary system | African | PB | 895 | 170 | 0.17 | PCR-RFLP |
| Hemissi[14] | 2021 | | Tunisia | bladder cancer | Tumor of urinary system | Caucasian | HB | 271 | 1123 | 0.884 | Taqman |
| Huang[15] | 2014 | | China | bladder cancer | Tumor of urinary system | Asian | HB | 159 | 797 | 0.226 | Taqman |
| Hung[16] | 2004 | | Italy | bladder cancer | Tumor of urinary system | Caucasian | HB | 201 | 260 | 0.046 | PCR-RFLP |
| Mandal[7] | 2012 | | India | bladder cancer | Tumor of urinary system | Caucasian | HB | 200 | 150 | 0.259 | PCR-RFLP |
| Moore[17] | 2004 | | Argentina | bladder cancer | Tumor of urinary system | Caucasian | PB | 106 | 214 | 0.21 | PCR-RFLP |
| Pandith[18] | 2011 | | India | bladder cancer | Tumor of urinary system | Asian | HB | 104 | 200 | 0.304 | PCR-RFLP |
| Park[19] | 2003 | | USA | bladder cancer | Tumor of urinary system | Caucasian | HB | 232 | 108 | 0.897 | PCR-RFLP |
| Sanyal[20] | 2004 | | Sweden | bladder cancer | Tumor of urinary system | Caucasian | PB | 299 | 120 | 0.79 | PCR-RFLP |
| Schulz[2] | 1997 | | Germany | bladder cancer | Tumor of urinary system | Caucasian | PB | 99 | 239 | 0.32 | PCR-RFLP |
| Terry[21] | 2005 | | USA | bladder cancer | Tumor of urinary system | Caucasian | HB | 235 | 124 | 0.18 | PCR-RFLP |
| Wang[22] | 2008 | | China | bladder cancer | Tumor of urinary system | Asian | HB | 300 | 260 | 0.76 | PCR-RFLP |
| Fu[23] | 2012 | | China | bladder cancer | Tumor of urinary system | Asian | HB | 99 | 214 | 0.16 | NA |
| Choi[24] | 2003 | | Korea | bladder cancer | Tumor of urinary system | Asian | HB | 177 | 170 | N/A | PCR-RFLP |
| Alexandrie[25] | 2004 | | Sweden | lung cancer | Respiratory tumors | Caucasian | PB | 524 | 162 | 0.71 | PCR-RFLP |
| Benhamou[26] | 2001 | | Finland | lung cancer | Respiratory tumors | Caucasian | HB | 150 | 167 | 0.88 | PCR-RFLP |
| Chan[27] | 2005 | | China | lung cancer | Respiratory tumors | Asian | HB | 74 | 405 | 0.67 | PCR-RFLP |
| Chen H[28] | 1999 | | USA | lung cancer | Respiratory tumors | Asian | PB | 109 | 121 | 0.48 | PCR-RFLP |
| Chen H[28] | 1999 | | USA | lung cancer | Respiratory tumors | Caucasian | PB | 135 | 100 | 0 | PCR |
| Cote[29] | 2009 | | USA | lung cancer | Respiratory tumors | Caucasian | PB | 387 | 597 | 0.17 | PCR-LDR |
| Cote[29] | 2009 | | USA | lung cancer | Respiratory tumors | African | PB | 113 | 640 | 0.26 | PCR-CTPP |
| Dai[30] | 2021 | | China | lung cancer | Respiratory tumors | Asian | HB | 98 | 109 | 0.98 | PCR-RFLP |
| Guo[31] | 2012 | | China | lung cancer | Respiratory tumors | Asian | HB | 681 | 145 | 0.86 | PCR-RFLP |
| Hamajima[5] | 2002 | | Japan | lung cancer | Respiratory tumors | Asian | HB | 192 | 227 | 0.46 | PCR-RFLP |
| Lan[32] | 2004 | | China | lung cancer | Respiratory tumors | Asian | PB | 119 | 152 | 0.62 | PCR-RFLP |
| Lewis[33] | 2001 | | UK | lung cancer | Respiratory tumors | Caucasian | HB | 82 | 136 | 0.27 | PCR-RFLP |
| Liang[34] | 2007 | | China | lung cancer | Respiratory tumors | Asian | HB | 227 | 50 | 0.1 | PCR |
| Liang GY[35] | 2004 | | China | lung cancer | Respiratory tumors | Asian | HB | 152 | 36 | 0.28 | PCR-RFLP |
| Lin P[36] | 2003 | | China | lung cancer | Respiratory tumors | Asian | HB | 198 | 683 | 0.84 | PCR-RFLP |
| Lin YH[37] | 2000 | | China | lung cancer | Respiratory tumors | Asian | HB | 95 | 107 | 0.56 | PCR-RFLP |
| Marco[38] | 2012 | | Thailand | lung cancer | Respiratory tumors | Asian | pb | 146 | 36 | 0.28 | PCR-RFLP |
| Saldivar[39] | 2005 | | USA | lung cancer | Respiratory tumors | African | PB | 36 | 597 | 0.19 | PCR-LDR |
| Saldivar[39] | 2005 | | USA | lung cancer | Respiratory tumors | Caucasian | PB | 107 | 267 | 0.62 | Taqman |
| Saldivar[39] | 2005 | | USA | lung cancer | Respiratory tumors | African | PB | 36 | 465 | 0.16 | PCR-RFLP |
| Shujie Guo[40] | 2012 | | China | lung cancer | Respiratory tumors | Asian | HB | 682 | 152 | 0.53 | PCR-RFLP |
| Sunaga[41] | 2002 | | Japan | lung cancer | Respiratory tumors | Asian | HB | 198 | 1096 | 0.93 | PCR-RFLP |
| Xu[42] | 2001 | | USA | lung cancer | Respiratory tumors | Caucasian | HB | 780 | 84 | 0.91 | PCR-RFLP |
| Xu[42] | 2001 | | USA | lung cancer | Respiratory tumors | Mixed | HB | 34 | 379 | 0.32 | PCR‑RFLP |
| Yang M[43] | 2007 | | Korea | lung cancer | Respiratory tumors | Asian | HB | 314 | 504 | 0.43 | PCR-RFLP |
| Yin L[44] | 2003 | | China | lung cancer | Respiratory tumors | Asian | HB | 74 | 177 | 0.91 | PCR-CTPP |
| Yin[45] | 2001 | | China | lung cancer | Respiratory tumors | Asian | HB | 84 | 640 | 0.08 | PCR-CTPP |
| YUZO[46] | 2017 | | Japan | lung cancer | Respiratory tumors | Asian | HB | 462 | 1689 | 0.054 | NA |
| Bock CH[47] | 2005 | | USA | lung cancer | Respiratory tumors | Caucasian | PB | 130 | 144 | 0.14 | PCR-RFLP |
| Bock CH[47] | 2005 | | USA | lung cancer | Respiratory tumors | African | PB | 31 | 29 | 0.26 | PCR-RFLP |
| Chen H[28] | 1999 | | USA | lung cancer | Respiratory tumors | Caucasian | PB | 83 | 102 | NA | PCR-RFLP |
| Eom SY[48] | 2009 | | Korea | lung cancer | Respiratory tumors | Asian | HB | 387 | 387 | NA | PCR-RFLP |
| Lawson KA[49] | 2005 | | Finland | lung cancer | Respiratory tumors | Caucasian | PB | 353 | 360 | NA | PCR-RFLP |
| Saldivar[39] | 2005 | | USA | lung cancer | Respiratory tumors | Caucasian | PB | 683 | 683 | NA | PCR-RFLP |
| Skuladottir H[50] | 2005 | | Denmark,Norway | lung cancer | Respiratory tumors | Caucasian | PB | 153 | 346 | NA | PCR-RFLP |
| Sorensen[51] | 2005 | | Denmark | lung cancer | Respiratory tumors | Caucasian | PB | 254 | 267 | NA | PCR-RFLP |
| Su[52] | 2009 | | China | lung cancer | Respiratory tumors | Asian | HB | 396 | 465 | NA | PCR-RFLP |
| Timofeeva M[53] | 2010 | | German | lung cancer | Respiratory tumors | Caucasian | PB | 617 | 1267 | 0.64 | PCR-RFLP |
| Wiencke JK[54] | 1997 | | USA | lung cancer | Respiratory tumors | Mixed | PB | 61 | 161 | 0.78 | Taqman |
| Wiencke JK[54] | 1997 | | USA | lung cancer | Respiratory tumors | African | PB | 116 | 136 | 0.91 | PCR |
| Franko[55] | 2018 | | Slovenia | malignant mesothelioma | Primary pleural tumor | Caucasian | PB | 158 | 201 | 0.34 | PCR-RFLP |
| Kang[56] | 2008 | | Korea | multiple myeloma | Hematological tumors | Asian | PB | 114 | 124 | 0.55 | PCR-RFLP |
| Lincz[57] | 2007 | | Australia | multiple myeloma | Hematological tumors | Caucasian | PB | 100 | 120 | 0.675 | PCR-RFLP |
| Maggini[58] | 2008 | | Italy | multiple myeloma | Hematological tumors | Caucasian | PB | 245 | 1035 | 0.76 | NA |
| Al-Dayel[59] | 2008 | | Saudi Arabia | lymphoma | Hematological tumors | Caucasian | PB | 150 | 214 | 0.89 | Taqman |
| Gra[60] | 2008 | | Russia | lymphoma | Hematological tumors | Caucasian | PB | 76 | 111 | 0.897 | Taqman |
| Hamajima[5] | 2002 | | Japan | lymphoma | Hematological tumors | Asian | HB | 108 | 295 | 0.831 | PCR-RFLP |
| Kim[61] | 2009 | | Korea | lymphoma | Hematological tumors | Asian | HB | 713 | 284 | 0.44 | Taqman |
| Pablo[62] | 2013 | | Spain | lymphoma | Hematological tumors | Caucasian | HB | 213 | 163 | 0.92 | Taqman |
| Yang[63] | 2005 | | China | leukemia | Hematological tumors | Asian | HB | 228 | 299 | 0.23 | PCR-RFLP |
| Yang[64] | 2008 | | China | leukemia | Hematological tumors | Asian | HB | 123 | 177 | 0.91 | PCR-CTPP |
| Andrade[65] | 2014 | | Brazil | leukemia | Hematological tumors | Caucasian | PB | 179 | 476 | 0.04 | PCR-RFLP |
| Begleiter[66] | 2009 | | Canada | leukemia | Hematological tumors | Caucasian | HB | 323 | 106 | 0.15 | PCR-RFLP |
| de Aguiar Goncalves[65] | 2012 | | Brazil | leukemia | Hematological tumors | Caucasian | HB | 546 | 175 | 0.1 | PCR-RFLP |
| Gra[60] | 2008 | | Russia | leukemia | Hematological tumors | Caucasian | PB | 83 | 155 | 0.2 | PCR-RFLP |
| Ouerhani[67] | 2013 | | Tunisia | leukemia | Hematological tumors | Asian | PB | 100 | 530 | 0.12 | PCR-RFLP |
| Seedhouse[68] | 2002 | | UK | leukemia | Hematological tumors | Caucasian | PB | 134 | 172 | 0.24 | PCR-RFLP |
| Voso[69] | 2007 | | Italy | leukemia | Hematological tumors | Caucasian | PB | 157 | 155 | NA | PCR-RFLP |
| Zhang[70] | 2005 | | China | leukemia | Hematological tumors | Asian | HB | 99 | 99 | 0.2 | Taqman |
| Hishida[71] | 2005 | | Japan | leukemia | Hematological tumors | Asian | HB | 51 | 476 | 0.199 | PCR-RFLP |
| Wu[72] | 2004 | | China | leukemia | Hematological tumors | Asian | HB | 61 | 63 | NA | PCR-RFLP |
| Begleiter[73] | 2005 | | USA/Canada | head and neck | Head-neck tumors | Caucasian | HB | 350 | 296 | 0.54 | PCR-RFLP |
| Benhamou[74] | 2001 | | Finland | head and neck | Head-neck tumors | Caucasian | HB | 250 | 1226 | 0.09 | PCR-RFLP |
| Harth[75] | 2008 | | Germany | head and neck | Head-neck tumors | Caucasian | HB | 295 | 504 | 0.43 | PCR-RFLP |
| Li[76] | 2005 | | USA | head and neck | Head-neck tumors | Caucasian | HB | 724 | 121 | 0.76 | PCR-RFLP |
| Siraj[77] | 2008 | | Saudi Arabia | head and neck | Head-neck tumors | Caucasian | PB | 49 | 167 | 0.81 | PCR-RFLP |
| Soucek[78] | 2010 | | Czech/Poland | head and neck | Head-neck tumors | Caucasian | HB | 116 | 526 | 0.41 | PCR-RFLP |
| Wu[79] | 2002 | | China | head and neck | Head-neck tumors | Asian | HB | 120 | 120 | NA | Taqman |
| Goode[80] | 2011 | | USA | ovarian cancer | Gynecological tumor | Caucasian | HB | 928 | 182 | 0.82 | PCR |
| Olson[81] | 2004 | | USA | ovarian cancer | Gynecological tumor | Caucasian | PB | 123 | 76 | 0.27 | PCR-RFLP |
| Gao[82] | 2010 | | China | endometrial carcinoma | Gynecological tumor | Asian | HB | 209 | 209 | 0.505 | PCR-RFLP |
| Jira Chansaenroj[83] | 2013 | | Thailand | cervical cancer | Gynecological tumor | Asian | HB | 56 | 32 | 0.08 | PCR |
| Nishino[84] | 2008 | | Japan | cervical cancer | Gynecological tumor | Asian | HB | 124 | 117 | 0.29 | PCR-RFLP |
| Niwa[85] | 2005 | | Japan | cervical cancer | Gynecological tumor | Asian | HB | 131 | 320 | 0.27 | PCR-CTPP |
| Thaís[86] | 2018 | | Brazil | cervical cancer | Gynecological tumor | Caucasian | HB | 86 | 299 | 0.06 | PCR-RFLP |
| Yang[87] | 2020 | | China | cervical cancer | Gynecological tumor | Asian | HB | 450 | 568 | 0.1 | PCR |
| Aston[88] | 2005 | | USA | breast cancer | Gynecological tumor | Caucasian | PB | 564 | 300 | 0.13 | PCR-RFLP |
| Hamajima[5] | 2002 | | Japan | breast cancer | Gynecological tumor | Asian | HB | 237 | 100 | 0.739 | PCR-CTPP |
| Hong[89] | 2007 | | USA | breast cancer | Gynecological tumor | Caucasian | PB | 496 | 1212 | 0.55 | PCR-RFLP |
| Hubackova[90] | 2009 | | Sulovak, Czech Republic | breast cancer | Gynecological tumor | Caucasian | HB | 52 | 640 | 0.17 | PCR-CTPP |
| Lajin[91] | 2013 | | Syria | breast cancer | Gynecological tumor | Caucasian | PB | 122 | 495 | 0.53 | Taqman |
| Menzel[92] | 2004 | | Austria | breast cancer | Gynecological tumor | Caucasian | PB | 218 | 53 | 0.827 | Taqman |
| Sarmanova[93] | 2004 | | Czech | breast cancer | Gynecological tumor | Caucasian | PB | 238 | 139 | 0.25 | ARMS-PCR |
| Siegelmann-Danieli[94] | 2002 | | USA | breast cancer | Gynecological tumor | Caucasian | PB | 346 | 424 | 0.17 | NA |
| Singh[95] | 2011 | | India | breast cancer | Gynecological tumor | Asian | PB | 200 | 310 | 0.58 | PCR-RFLP |
| Miluse[96] | 2011 | | Czech Republic | breast cancer | Gynecological tumor | Caucasian | HB | 321 | 235 | 0.87 | PCR-RFLP |
| Chen[97] | 2015 | | China | breast cancer | Gynecological tumor | Asian | HB | 232 | 200 | 0.05 | PCR-RFLP |
| Osamu[98] | 2015 | | Japan | cervical cancer | Gynecological tumor | Asian | HB | 142 | 54 | NA | PCR |
| Wang[99] | 2015 | | China | breast cancer | Gynecological tumor | Asian | HB | 248 | 284 | NA | PCR |
| Bartsch[100] | 1998 | | USA/Europe | pancreatic cancer | Digestive cancer | Caucasian | HB | 81 | 265 | 0.93 | Taqman |
| Mohelnikova-Duchonova[101] | 2011 | | Czech | pancreatic cancer | Digestive cancer | Caucasian | HB | 235 | 235 | 0.8 | PCR-RFLP |
| Mohelnikova-Duchonova[101] | 2011 | | Sulovak, Czech Republic | pancreatic cancer | Digestive cancer | Caucasian | HB | 265 | 59 | 0.571 | PCR-RFLP |
| Fei Liu[102] | 2012 | | China | hepatocellular carcinoma | Digestive cancer | Asian | HB | 476 | 526 | 0.07 | NA |
| Hikmet Akkiz[103] | 2010 | | turkey | hepatocellular carcinoma | Digestive cancer | Caucasian | HB | 167 | 163 | 0.93 | PCR-RFLP |
| Tan[104] | 2012 | | China | hepatocellular carcinoma | Digestive cancer | Asian | HB | 400 | 400 | 0.07 | TaqMan-MGB |
| Wang[105] | 2013 | | China | hepatocellular carcinoma | Digestive cancer | Asian | HB | 282 | 274 | 0.06 | TaqMan-MGB |
| Akkiz[103] | 2010 | | Turkey | hepatocellular carcinoma | Digestive cancer | Caucasian | HB | 167 | 366 | 0.94 | PCR-RFLP |
| Liu[102] | 2013 | | China | hepatocellular carcinoma | Digestive cancer | Asian | HB | 476 | 172 | 0.24 | PCR-RFLP |
| Gui[106] | 2011 | | China | hepatocellular carcinoma | Digestive cancer | Asian | HB | 34 | 41 | 0.04 | PCR-RFLP |
| Zhang[107] | 2014 | | China | gastric cancer | Digestive cancer | Asian | HB | 297 | 300 | 0.298 | NA |
| Chen D[108] | 2011 | | China | gastric cancer | Digestive cancer | Asian | PB | 334 | 334 | 0.002 | PCR-RFLP |
| Chen D[109] | 2007 | | China | gastric cancer | Digestive cancer | Asian | PB | 112 | 104 | 0.07 | PCR-RFLP |
| Hamajima[5] | 2002 | | Japan | gastric cancer | Digestive cancer | Asian | HB | 143 | 640 | 0.07 | PCR-CTPP |
| Hu[110] | 2014 | | China | gastric cancer | Digestive cancer | Asian | HB | 441 | 597 | 0.19 | PCR-LDR |
| Li[111] | 2004 | | China | gastric cancer | Digestive cancer | Asian | HB | 124 | 165 | 0.22 | PCR-RFLP |
| Malik[96] | 2011 | | India | gastric cancer | Digestive cancer | Asian | HB | 108 | 195 | 0.3 | PCR-RFLP |
| Ren[112] | 2006 | | China | gastric cancer | Digestive cancer | Asian | PB | 80 | 80 | 0.26 | PCR-RFLP |
| Sarbia[113] | 2003 | | Germany | gastric cancer | Digestive cancer | Caucasian | HB | 320 | 252 | 0.6 | PCR-RFLP |
| Zhang J. H.[114] | 2003 | | China | gastric cancer | Digestive cancer | Asian | HB | 124 | 165 | 0.39 | PCR |
| Gui[106] | 2011 | | China | familial aggregation liver cancer | Digestive cancer | Asian | HB | 34 | 32 | 0.001 | PCR-RFLP |
| di Martino[115] | 2007 | | UK | esophageal cancer | Digestive cancer | Caucasian | HB | 141 | 93 | 0.98 | PCR-RFLP |
| Feng[116] | 2008 | | China | esophageal cancer | Digestive cancer | Asian | HB | 201 | 201 | 0.14 | PCR-RFLP |
| Freriksen[117] | 2014 | | Netherlands | esophageal cancer | Digestive cancer | Caucasian | HB | 467 | 474 | 0.13 | PCR-RFLP |
| Hamajima[5] | 2002 | | Japan | esophageal cancer | Digestive cancer | Asian | HB | 102 | 640 | 0.07 | PCR-CTPP |
| Jun[118] | 2014 | | China | esophageal cancer | Digestive cancer | Asian | HB | 600 | 651 | 0.142 | NA |
| Li[119] | 2019 | | China | esophageal cancer | Digestive cancer | Asian | HB | 122 | 123 | 0 | PCR |
| Malik[120] | 2012 | | India | esophageal cancer | Digestive cancer | Asian | HB | 135 | 195 | 0.3 | PCR-RFLP |
| Marjani[121] | 2010 | | Iran | esophageal cancer | Digestive cancer | Caucasian | HB | 93 | 50 | 0.46 | PCR-RFLP |
| Sarbia[113] | 2003 | | Germany | esophageal cancer | Digestive cancer | Caucasian | HB | 61 | 252 | 0.6 | PCR-RFLP |
| Umar[122] | 2012 | | India | esophageal cancer | Digestive cancer | Asian | HB | 200 | 200 | 0.86 | PCR-RFLP |
| von Rahden[123] | 2005 | | Germany | esophageal cancer | Digestive cancer | Caucasian | HB | 140 | 260 | 0.16 | PCR-RFLP |
| Yin[118] | 2014 | | China | esophageal cancer | Digestive cancer | Asian | HB | 600 | 651 | 0.14 | PCR |
| Zhang[124] | 2006 | | China | esophageal cancer | Digestive cancer | Asian | PB | 96 | 192 | 0.72 | PCR-RFLP |
| Zhang J.[125] | 2003 | | China | esophageal cancer | Digestive cancer | Asian | HB | 193 | 141 | 0.39 | PCR-RFLP |
| Zhang J. H.[114] | 2003 | | China | esophageal cancer | Digestive cancer | Asian | HB | 193 | 165 | 0.39 | PCR |
| Zhou[126] | 2006 | | China | esophageal cancer | Digestive cancer | Asian | HB | 96 | 192 | 0.72 | PCR-RFLP |
| Martino[115] | 2007 | | UK | esophageal cancer | Digestive cancer | Caucasian | HB | 144 | 93 | 0.24 | PCR-RFLP |
| Rahden[123] | 2004 | | German | esophageal cancer | Digestive cancer | Caucasian | HB | 140 | 260 | 0.166 | PCR-RFLP |
| Zhang J.[125] | 2003 | | German | esophageal cancer | Digestive cancer | Caucasian | HB | 257 | 252 | 0.93 | PCR-RFLP |
| Begleiter[127] | 2006 | | Canada | colorectal cancer | Digestive cancer | Caucasian | PB | 369 | 123 | 0.32 | NA |
| Dai[128] | 2004 | | China | colorectal cancer | Digestive cancer | Asian | HB | 101 | 415 | 0.94 | PCR-RFLP |
| Freriksen[117] | 2014 | | Netherlands | colorectal cancer | Digestive cancer | Caucasian | HB | 541 | 103 | 0.749 | PCR-RFLP |
| Hamachi[129] | 2013 | | Japan | colorectal cancer | Digestive cancer | Asian | HB | 455 | 542 | 0.42 | PCR-RFLP |
| Hamajima[5] | 2002 | | Japan | colorectal cancer | Digestive cancer | Asian | HB | 146 | 1052 | 0.76 | PCR-RFLP |
| Harth[130] | 2000 | | Germany | colorectal cancer | Digestive cancer | Caucasian | PB | 323 | 640 | 0.07 | PCR-CTPP |
| Hlavata[131] | 2010 | | Czech | colorectal cancer | Digestive cancer | Caucasian | PB | 86 | 205 | 0.79 | PCR-RFLP |
| Hou[132] | 2005 | | USA | colorectal cancer | Digestive cancer | Caucasian | PB | 700 | 160 | 0.44 | Taqman |
| Lin[133] | 2010 | | China | colorectal cancer | Digestive cancer | Asian | HB | 148 | 708 | 0.007 | Taqman |
| Mitrou[134] | 2007 | | UK | colorectal cancer | Digestive cancer | Caucasian | PB | 889 | 148 | 0.10 | PCR-RFLP |
| Nisa[135] | 2010 | | Japan | colorectal cancer | Digestive cancer | Asian | PB | 684 | 936 | 0.58 | PCR-RFLP |
| Northwood[136] | 2010 | | England | colorectal cancer | Digestive cancer | Caucasian | HB | 495 | 777 | 0.06 | PCR-RFLP |
| Peng[137] | 2008 | | China | colorectal cancer | Digestive cancer | Asian | HB | 286 | 495 | 0.84 | Taqman |
| Peng[136] | 2013 | | China | colorectal cancer | Digestive cancer | Asian | HB | 672 | 286 | 0.32 | PCR-RFLP |
| Sachse[138] | 2002 | | UK | colorectal cancer | Digestive cancer | Caucasian | PB | 500 | 672 | 0.24 | PCR-RFLP |
| Sameer[139] | 2010 | | India | colorectal cancer | Digestive cancer | Asian | HB | 311 | 593 | 0.55 | PCR-RFLP |
| Su[140] | 2012 | | China | colorectal cancer | Digestive cancer | Asian | HB | 76 | 296 | 0.79 | PCR-RFLP |
| Tijhuis[141] | 2008 | | Netherlands | colorectal cancer | Digestive cancer | Caucasian | HB | 737 | 160 | 0.01 | PCR-RFLP |
| van der Logt[142] | 2006 | | Netherlands | colorectal cancer | Digestive cancer | Caucasian | PB | 298 | 698 | 0.28 | Taqman |
| Liu[143] | 2002 | | China | colorectal cancer | Digestive cancer | Asian | HB | 51 | 349 | 0.45 | PCR-RFLP |
| Li[144] | 2004 | | China | cardiac carcinoma | Digestive cancer | Asian | HB | 165 | 124 | 0.24 | PCR-RFLP |
|  |  | |  |  |  |  |  |  |  |  |  |

HB: hospital-based; PB: population-based; SOC; source of control; PCR-RFLP: polymerase chain reaction followed by restriction fragment length polymorphism; PCR-LDR: polymerase chain reaction by ligase detection reaction; HWE: Hardy-Weinberg equilibrium of control group; NA: not available; PCR-CTPP: PCR-confronting two-pair primers.

Reference

[1] S. Longuemaux, C. Delomenie, C. Gallou, A. Mejean, M. Vincent-Viry, R. Bouvier, D. Droz, R. Krishnamoorthy, M.M. Galteau, C. Junien, C. Beroud, and J.M. Dupret, Candidate genetic modifiers of individual susceptibility to renal cell carcinoma: a study of polymorphic human xenobiotic-metabolizing enzymes. Cancer Res 59 (1999) 2903-8.

[2] W.A. Schulz, A. Krummeck, I. Rösinger, P. Eickelmann, C. Neuhaus, T. Ebert, B.J. Schmitz-Dräger, and H. Sies, Increased frequency of a null-allele for NAD(P)H: quinone oxidoreductase in patients with urological malignancies. Pharmacogenetics 7 (1997) 235-9.

[3] H.A. Ergen, U. Gormus, F. Narter, U. Zeybek, S. Bulgurcuoglu, and T. Isbir, Investigation of NAD(P)H:quinone oxidoreductase 1 (NQO1) C609T polymorphism in prostate cancer. Anticancer Res 27 (2007) 4107-10.

[4] Z. Gong, M.E. Platek, C. Till, P.J. Goodman, C.M. Tangen, E.A. Platz, M.L. Neuhouser, I.M. Thompson, R.M. Santella, and C.B. Ambrosone, Associations Between Polymorphisms in Genes Related to Oxidative Stress and DNA Repair, Interactions With Serum Antioxidants, and Prostate Cancer Risk: Results From the Prostate Cancer Prevention Trial. Front Oncol 11 (2021) 808715.

[5] N. Hamajima, K. Matsuo, H. Iwata, M. Shinoda, Y. Yamamura, T. Kato, S. Hatooka, T. Mitsudomi, M. Suyama, Y. Kagami, M. Ogura, M. Ando, Y. Sugimura, and K. Tajima, NAD(P)H: quinone oxidoreductase 1 (NQO1) C609T polymorphism and the risk of eight cancers for Japanese. Int J Clin Oncol 7 (2002) 103-8.

[6] L.-L.i.L. Jing-Xian Z, Ya-Wen W, NQO1 C609T gene polymorphism associated with an increased risk of pros tate cancer cognitive dysfunction. Chin J Behav Med and Brain Sci (2011) 698– 700.

[7] R.K. Mandal, K. Nissar, and R.D. Mittal, Genetic variants in metabolizing genes NQO1, NQO2, MTHFR and risk of prostate cancer: a study from North India. Mol Biol Rep 39 (2012) 11145-52.

[8] A. Steinbrecher, S. Rohrmann, M. Timofeeva, A. Risch, E. Jansen, and J. Linseisen, Dietary glucosinolate intake, polymorphisms in selected biotransformation enzymes, and risk of prostate cancer. Cancer Epidemiol Biomarkers Prev 19 (2010) 135-43.

[9] M. Steiner, M. Hillenbrand, M. Borkowsi, H. Seiter, and P. Schuff-Werner, 609 C --> T polymorphism in NAD(P)H:quinone oxidoreductase gene in patients with prostatic adenocarcinoma or benign prostatic hyperplasia. Cancer Lett 135 (1999) 67-71.

[10] C.G. Stoehr, E. Nolte, S. Wach, W.F. Wieland, F. Hofstaedter, A. Hartmann, and R. Stoehr, NAD(P)H:quinone oxidoreductase 1 (NQO1) P187S polymorphism and prostate cancer risk in Caucasians. Int J Mol Sci 13 (2012) 10959-69.

[11] K. Broberg, J. Bjork, K. Paulsson, M. Hoglund, and M. Albin, Constitutional short telomeres are strong genetic susceptibility markers for bladder cancer. Carcinogenesis 26 (2005) 1263-71.

[12] J.D. Figueroa, N. Malats, M. Garcia-Closas, F.X. Real, D. Silverman, M. Kogevinas, S. Chanock, R. Welch, M. Dosemeci, Q. Lan, A. Tardon, C. Serra, A. Carrato, R. Garcia-Closas, G. Castano-Vinyals, and N. Rothman, Bladder cancer risk and genetic variation in AKR1C3 and other metabolizing genes. Carcinogenesis 29 (2008) 1955-62.

[13] D. Goerlitz, S. Amr, C. Dash, D.A. Saleh, M. El Daly, M. Abdel-Hamid, S. El Kafrawy, T. Hifnawy, S. Ezzat, M.A. Abdel-Aziz, H. Khaled, Y.L. Zheng, N. Mikhail, and C.A. Loffredo, Genetic polymorphisms in NQO1 and SOD2: interactions with smoking, schistosoma infection, and bladder cancer risk in Egypt. Urol Oncol 32 (2014) 47 e15-20.

[14] I. Hemissi, H. Ayed, Z. Naimi, K. Meddeb, M. Ayadi, S. Zouari, S. Zaghbib, E. Talbi, M. Chebil, and S. Ouerhani, Polymorphisms in NQO1 and MPO genes and risk for bladder cancer in Tunisian population. Mol Genet Genomic Med 9 (2021) e1819.

[15] Z.M. Huang, H.A. Chen, Y.T. Chiang, C.H. Shen, M.C. Tung, and G.D. Juang, Association of polymorphisms in iNOS and NQO1 with bladder cancer risk in cigarette smokers. J Chin Med Assoc 77 (2014) 83-8.

[16] R.J. Hung, P. Boffetta, P. Brennan, C. Malaveille, U. Gelatti, D. Placidi, A. Carta, A. Hautefeuille, and S. Porru, Genetic polymorphisms of MPO, COMT, MnSOD, NQO1, interactions with environmental exposures and bladder cancer risk. Carcinogenesis 25 (2004) 973-8.

[17] L.E. Moore, J.K. Wiencke, M.N. Bates, S. Zheng, O.A. Rey, and A.H. Smith, Investigation of genetic polymorphisms and smoking in a bladder cancer case-control study in Argentina. Cancer Lett 211 (2004) 199-207.

[18] A.A. Pandith, N.P. Khan, Z.A. Shah, A.M. Shah, S.M. Wani, and M.A. Siddiqi, Association of bladder cancer risk with an NAD(P)H:quinone oxidoreductase polymorphism in an ethnic Kashmiri population. Biochem Genet 49 (2011) 417-26.

[19] S.J. Park, H. Zhao, M.R. Spitz, H.B. Grossman, and X. Wu, An association between NQO1 genetic polymorphism and risk of bladder cancer. Mutat Res 536 (2003) 131-7.

[20] S. Sanyal, F. Festa, S. Sakano, Z. Zhang, G. Steineck, U. Norming, H. Wijkstrom, P. Larsson, R. Kumar, and K. Hemminki, Polymorphisms in DNA repair and metabolic genes in bladder cancer. Carcinogenesis 25 (2004) 729-34.

[21] P.D. Terry, D.M. Umbach, and J.A. Taylor, No association between SOD2 or NQO1 genotypes and risk of bladder cancer. Cancer Epidemiol Biomarkers Prev 14 (2005) 753-4.

[22] Y.H. Wang, Y.H. Lee, P.T. Tseng, C.H. Shen, and H.Y. Chiou, Human NAD(P)H:quinone oxidoreductase 1 (NQO1) and sulfotransferase 1A1 (SULT1A1) polymorphisms and urothelial cancer risk in Taiwan. J Cancer Res Clin Oncol 134 (2008) 203-9.

[23] C.B. Fu J, Relationship between genetic polymor phisms of CYP1A1, NQO1 and EPHX1 and susceptibility to bladder cancer. Chin J Cancer Prev Treat 18 (2012) 561-564.

[24] J.Y. Choi, K.M. Lee, S.H. Cho, S.W. Kim, H.Y. Choi, S.Y. Lee, H.J. Im, K.J. Yoon, H. Choi, I. Choi, A. Hirvonen, R.B. Hayes, and D. Kang, CYP2E1 and NQO1 genotypes, smoking and bladder cancer. Pharmacogenetics 13 (2003) 349-55.

[25] A.K. Alexandrie, F. Nyberg, M. Warholm, and A. Rannug, Influence of CYP1A1, GSTM1, GSTT1, and NQO1 genotypes and cumulative smoking dose on lung cancer risk in a Swedish population. Cancer Epidemiol Biomarkers Prev 13 (2004) 908-14.

[26] C. Bouchardy, S. Benhamou, N. Jourenkova, P. Dayer, and A. Hirvonen, Metabolic genetic polymorphisms and susceptibility to lung cancer. Lung Cancer 32 (2001) 109-12.

[27] E.C. Chan, S.Y. Lam, K.H. Fu, and Y.L. Kwong, Polymorphisms of the GSTM1, GSTP1, MPO, XRCC1, and NQO1 genes in Chinese patients with non-small cell lung cancers: relationship with aberrant promoter methylation of the CDKN2A and RARB genes. Cancer Genet Cytogenet 162 (2005) 10-20.

[28] H. Chen, A. Lum, A. Seifried, L.R. Wilkens, and L. Le Marchand, Association of the NAD(P)H:quinone oxidoreductase 609C-->T polymorphism with a decreased lung cancer risk. Cancer Res 59 (1999) 3045-8.

[29] M.L. Cote, W. Yoo, A.S. Wenzlaff, G.M. Prysak, S.K. Santer, G.B. Claeys, A.L. Van Dyke, S.J. Land, and A.G. Schwartz, Tobacco and estrogen metabolic polymorphisms and risk of non-small cell lung cancer in women. Carcinogenesis 30 (2009) 626-35.

[30] D.J. Zhibi, Correlation betweenpolymorphisms of CD44 and NQO1 gene andlung cancer susceptibility and prognosis of chemotherapy.

Chinese Journal of Surgical Oncology 13 (2021) 283-286.

[31] S. Guo, X. Li, M. Gao, H. Kong, Y. Li, M. Gu, X. Dong, and W. Niu, Synergistic association of PTGS2 and CYP2E1 genetic polymorphisms with lung cancer risk in northeastern Chinese. PLoS One 7 (2012) e39814.

[32] Q. Lan, J.L. Mumford, M. Shen, D.M. Demarini, M.R. Bonner, X. He, M. Yeager, R. Welch, S. Chanock, L. Tian, R.S. Chapman, T. Zheng, P. Keohavong, N. Caporaso, and N. Rothman, Oxidative damage-related genes AKR1C3 and OGG1 modulate risks for lung cancer due to exposure to PAH-rich coal combustion emissions. Carcinogenesis 25 (2004) 2177-81.

[33] S.J. Lewis, N.M. Cherry, R.M. Niven, P.V. Barber, and A.C. Povey, Polymorphisms in the NAD(P)H: quinone oxidoreductase gene and small cell lung cancer risk in a UK population. Lung Cancer 34 (2001) 177-83.

[34] L.G.-y.Y.-p. Li-hong, Case-only Study on the Gene-environmental Interaction Related with Lung Cancer Risk.

JOURNAL OF ENVIRONMENTAL AND OCCUPATIONAL MEDICINE 23 (2006) 449-453.

[35] G.Y. Liang, Y.P. Pu, and L.H. Yin, [Studies of the genes related to lung cancer susceptibility in Nanjing Han population, China]. Yi Chuan 26 (2004) 584-8.

[36] P. Lin, Y.M. Hsueh, J.L. Ko, Y.F. Liang, K.J. Tsai, and C.Y. Chen, Analysis of NQO1, GSTP1, and MnSOD genetic polymorphisms on lung cancer risk in Taiwan. Lung Cancer 40 (2003) 123-9.

[37] L.X. Lin Y, Shao M, Liang Y, Li Y, NAD(P)H: quinone oxidoredictase gene polymorphism and susceptibility of lung cancer. J Thorac Oncol 6 (2000) 1826–1832.

[38] M. Peluso, P. Srivatanakul, A. Jedpiyawongse, S. Sangrajrang, A. Munnia, S. Piro, M. Ceppi, P. Boffetta, R.W. Godschalk, and F.J. van Schooten, Aromatic DNA adducts and number of lung cancer risk alleles in Map-Ta-Phut Industrial Estate workers and nearby residents. Mutagenesis 28 (2013) 57-63.

[39] S.J. Saldivar, Y. Wang, H. Zhao, L. Shao, J. Lin, M.R. Spitz, and X. Wu, An association between a NQO1 genetic polymorphism and risk of lung cancer. Mutat Res 582 (2005) 71-8.

[40] S. Guo, M. Gao, X. Li, Y. Li, S. Chu, D. Zhu, and W. Niu, Lack of association between NADPH quinone oxidoreductase 1 (NQO1) gene C609T polymorphism and lung cancer: a case-control study and a meta-analysis. PLoS One 7 (2012) e47939.

[41] N. Sunaga, T. Kohno, N. Yanagitani, H. Sugimura, H. Kunitoh, T. Tamura, Y. Takei, S. Tsuchiya, R. Saito, and J. Yokota, Contribution of the NQO1 and GSTT1 polymorphisms to lung adenocarcinoma susceptibility. Cancer Epidemiol Biomarkers Prev 11 (2002) 730-8.

[42] L.L. Xu, J.C. Wain, D.P. Miller, S.W. Thurston, L. Su, T.J. Lynch, and D.C. Christiani, The NAD(P)H:quinone oxidoreductase 1 gene polymorphism and lung cancer: differential susceptibility based on smoking behavior. Cancer Epidemiol Biomarkers Prev 10 (2001) 303-9.

[43] M. Yang, Y. Choi, B. Hwangbo, and J.S. Lee, Combined effects of genetic polymorphisms in six selected genes on lung cancer susceptibility. Lung Cancer 57 (2007) 135-42.

[44] Y.P. L Yin 1, T Y Liu, Y H Tung, K W Chen, P Lin, A Study on Relationship between Susceptibility to Lung Cancer and Polymorphisms of NQO1,CYP1A1,mEH Genes.

CHINESE JOURNAL OF ENVIRONMENTAL & OCCUPATIONAL MEDICINE 20 (2003) 22-25.

[45] L. Yin, Y. Pu, T.Y. Liu, Y.H. Tung, K.W. Chen, and P. Lin, Genetic polymorphisms of NAD(P)H quinone oxidoreductase, CYP1A1 and microsomal epoxide hydrolase and lung cancer risk in Nanjing, China. Lung Cancer 33 (2001) 133-41.

[46] Y. Yamamoto, C. Kiyohara, S. Suetsugu-Ogata, N. Hamada, and Y. Nakanishi, Biological interaction of cigarette smoking on the association between genetic polymorphisms involved in inflammation and the risk of lung cancer: A case-control study in Japan. Oncol Lett 13 (2017) 3873-3881.

[47] C.H. Bock, A.S. Wenzlaff, M.L. Cote, S.J. Land, and A.G. Schwartz, NQO1 T allele associated with decreased risk of later age at diagnosis lung cancer among never smokers: results from a population-based study. Carcinogenesis 26 (2005) 381-6.

[48] S.Y. Eom, Y.W. Zhang, S.H. Kim, K.H. Choe, K.Y. Lee, J.D. Park, Y.C. Hong, Y.D. Kim, J.W. Kang, and H. Kim, Influence of NQO1, ALDH2, and CYP2E1 genetic polymorphisms, smoking, and alcohol drinking on the risk of lung cancer in Koreans. Cancer Causes Control 20 (2009) 137-45.

[49] K.A. Lawson, K. Woodson, J. Virtamo, and D. Albanes, Association of the NAD(P)H:quinone oxidoreductase (NQO1) 609C->T polymorphism with lung cancer risk among male smokers. Cancer Epidemiol Biomarkers Prev 14 (2005) 2275-6.

[50] H. Skuladottir, H. Autrup, J. Autrup, A. Tjoenneland, K. Overvad, D. Ryberg, A. Haugen, and J.H. Olsen, Polymorphisms in genes involved in xenobiotic metabolism and lung cancer risk under the age of 60 years. A pooled study of lung cancer patients in Denmark and Norway. Lung Cancer 48 (2005) 187-99.

[51] M. Sorensen, H. Autrup, A. Tjonneland, K. Overvad, and O. Raaschou-Nielsen, Genetic polymorphisms in CYP1B1, GSTA1, NQO1 and NAT2 and the risk of lung cancer. Cancer Lett 221 (2005) 185-90.

[52] S.J.N. Run, Association between polymorphisms of phase Ⅱ metabolic gene NQO1 and susceptibility to lung cancer. CHINESE JOURNAL OF PUBLIC HEALTH 25 (2009) 458-460.

[53] M. Timofeeva, S. Kropp, W. Sauter, L. Beckmann, A. Rosenberger, T. Illig, B. Jager, K. Mittelstrass, H. Dienemann, L. Consortium, H. Bartsch, H. Bickeboller, J. Chang-Claude, A. Risch, and H.E. Wichmann, Genetic polymorphisms of MPO, GSTT1, GSTM1, GSTP1, EPHX1 and NQO1 as risk factors of early-onset lung cancer. Int J Cancer 127 (2010) 1547-61.

[54] J.K. Wiencke, M.R. Spitz, A. McMillan, and K.T. Kelsey, Lung cancer in Mexican-Americans and African-Americans is associated with the wild-type genotype of the NAD(P)H: quinone oxidoreductase polymorphism. Cancer Epidemiol Biomarkers Prev 6 (1997) 87-92.

[55] A. Franko, N. Kotnik, K. Goricar, V. Kovac, M. Dodic-Fikfak, and V. Dolzan, The Influence of Genetic Variability on the Risk of Developing Malignant Mesothelioma. Radiol Oncol 52 (2018) 105-111.

[56] S.H. Kang, T.Y. Kim, H.Y. Kim, J.H. Yoon, H.I. Cho, S.S. Yoon, D.H. Kang, C.W. Suh, J.H. Lee, and D.S. Lee, Protective role of CYP1A1*2A in the development of multiple myeloma. Acta Haematol 119 (2008) 60-4.

[57] L.F. Lincz, F.E. Scorgie, R. Robertson, and A. Enno, Genetic variations in benzene metabolism and susceptibility to multiple myeloma. Leuk Res 31 (2007) 759-63.

[58] V. Maggini, G. Buda, S. Galimberti, A. Martino, E. Orciuolo, F. Morabito, R. Bono, R. Barale, M. Petrini, and A.M. Rossi, Lack of association of NQO1 and GSTP1 polymorphisms with multiple myeloma risk. Leuk Res 32 (2008) 988-90.

[59] F. Al-Dayel, M. Al-Rasheed, M. Ibrahim, R. Bu, P. Bavi, J. Abubaker, N. Al-Jomah, G.H. Mohamed, A. Moorji, S. Uddin, A.K. Siraj, and K. Al-Kuraya, Polymorphisms of drug-metabolizing enzymes CYP1A1, GSTT and GSTP contribute to the development of diffuse large B-cell lymphoma risk in the Saudi Arabian population. Leuk Lymphoma 49 (2008) 122-9.

[60] O.A. Gra, A.S. Glotov, E.A. Nikitin, O.S. Glotov, V.E. Kuznetsova, A.V. Chudinov, A.B. Sudarikov, and T.V. Nasedkina, Polymorphisms in xenobiotic-metabolizing genes and the risk of chronic lymphocytic leukemia and non-Hodgkin's lymphoma in adult Russian patients. Am J Hematol 83 (2008) 279-87.

[61] H.N. Kim, N.Y. Kim, L. Yu, Y.K. Kim, I.K. Lee, D.H. Yang, J.J. Lee, M.H. Shin, K.S. Park, J.S. Choi, and H.J. Kim, Polymorphisms of drug-metabolizing genes and risk of non-Hodgkin lymphoma. Am J Hematol 84 (2009) 821-5.

[62] P. Conesa-Zamora, J. Ruiz-Cosano, D. Torres-Moreno, I. Español, M.D. Gutiérrez-Meca, J. Trujillo-Santos, E. Pérez-Ceballos, R. González-Conejero, J. Corral, V. Vicente, and M. Pérez-Guillermo, Polymorphisms in xenobiotic metabolizing genes (EPHX1, NQO1 and PON1) in lymphoma susceptibility: a case control study. BMC Cancer 13 (2013) 228.

[63] Y.L.Z.Y.Z.M.-r.X. Zhi-jian, Relationship between GSTT1,GSTM1 and NQO1 gene polymorphism and acute myeloid leukemia and recurrent chromosome translocations. NATIONAL MEDICAL JOURNAL OF CHINA 85 (2005) 2312-2316.

[64] Z.Z.-Q.L.Y.Y.-H.L.L.J.-X.X.-F. Zhi-Jian, Relationship between NQO1C609T,RAD51 G135C,XRCC3 C241T Single Nucleotide Polymorphisms and Acute Lymphoblastic Leukemia.

JOURNAL OF EXPERIMENTAL HEMATOLOGY 17 (2009) 523-528.

[65] T.C. Barbosa, F.G. Andrade, B.A. Lopes, C.F. de Andrade, M.B. Mansur, M. Emerenciano, and M.S. Pombo-de-Oliveira, Impact of mutations in FLT3, PTPN11 and RAS genes on the overall survival of pediatric B cell precursor acute lymphoblastic leukemia in Brazil. Leuk Lymphoma 55 (2014) 1501-9.

[66] A. Begleiter, D. Hewitt, S.B. Gibson, and J.B. Johnston, Investigation of an NQO1 polymorphism as a possible risk and prognostic factor for chronic lymphocytic leukemia. Leuk Res 33 (2009) 74-81.

[67] S. Ouerhani, N. Cherif, I. Bahri, I. Safra, S. Menif, and S. Abbes, Genetic polymorphisms of NQO1, CYP1A1 and TPMT and susceptibility to acute lymphoblastic leukemia in a Tunisian population. Mol Biol Rep 40 (2013) 1307-14.

[68] C. Seedhouse, R. Bainton, M. Lewis, A. Harding, N. Russell, and E. Das-Gupta, The genotype distribution of the XRCC1 gene indicates a role for base excision repair in the development of therapy-related acute myeloblastic leukemia. Blood 100 (2002) 3761-6.

[69] M.T. Voso, E. Fabiani, F. D'Alo, F. Guidi, A. Di Ruscio, S. Sica, L. Pagano, M. Greco, S. Hohaus, and G. Leone, Increased risk of acute myeloid leukaemia due to polymorphisms in detoxification and DNA repair enzymes. Ann Oncol 18 (2007) 1523-8.

[70] Z.J.Y.L.F. Ji, Study on the relationship between genetic polymorphism and susceptibility for adult acute leukemia. TUMOR 25 (2005) 346-350.

[71] A. Hishida, S. Terakura, N. Emi, K. Yamamoto, M. Murata, K. Nishio, Y. Sekido, T. Niwa, N. Hamajima, and T. Naoe, GSTT1 and GSTM1 deletions, NQO1 C609T polymorphism and risk of chronic myelogenous leukemia in Japanese. Asian Pac J Cancer Prev 6 (2005) 251-5.

[72] W. Yuxia, Study on environmental risk factors and toxicant metabolizing enzyme gene polymorphism in childhood leukemia, 2003.

[73] A. Begleiter, A. Norman, D. Leitao, T. Cabral, D. Hewitt, S. Pan, J.R. Grandis, J.M. Siegfried, S. El-Sayed, D. Sutherland, D.A. Ross, and P.D. Kerr, Role of NQO1 polymorphisms as risk factors for squamous cell carcinoma of the head and neck. Oral Oncol 41 (2005) 927-33.

[74] S. Benhamou Anu Voho Christine Bouchardy Katja Mitrunen Pierre Dayer Ari Hirvonen, Role of NAD(P)H:quinone oxidoreductase polymorphism at codon 187 in susceptibility to lung, laryngeal and oral/pharyngeal cancers. Biomarkers 6 (2001) 440-7.

[75] V. Harth, M. Schafer, J. Abel, L. Maintz, T. Neuhaus, M. Besuden, R. Primke, A. Wilkesmann, R. Thier, H. Vetter, Y.D. Ko, T. Bruning, H.M. Bolt, and K. Ickstadt, Head and neck squamous-cell cancer and its association with polymorphic enzymes of xenobiotic metabolism and repair. J Toxicol Environ Health A 71 (2008) 887-97.

[76] G. Li, Z. Liu, E.M. Sturgis, R.M. Chamberlain, M.R. Spitz, and Q. Wei, CYP2E1 G1532C, NQO1 Pro187Ser, and CYP1B1 Val432Leu polymorphisms are not associated with risk of squamous cell carcinoma of the head and neck. Cancer Epidemiol Biomarkers Prev 14 (2005) 1034-6.

[77] A.K. Siraj, M. Al-Rasheed, M. Ibrahim, K. Siddiqui, F. Al-Dayel, O. Al-Sanea, S. Uddin, and K. Al-Kuraya, RAD52 polymorphisms contribute to the development of papillary thyroid cancer susceptibility in Middle Eastern population. J Endocrinol Invest 31 (2008) 893-9.

[78] P. Soucek, S. Susova, B. Mohelnikova-Duchonova, J. Gromadzinska, A. Moraviec-Sztandera, P. Vodicka, and L. Vodickova, Polymorphisms in metabolizing enzymes and the risk of head and neck squamous cell carcinoma in the Slavic population of the central Europe. Neoplasma 57 (2010) 415-21.

[79] Wu-DeHua, Quinone oxidoreductase gene polymorphism and genetic susceptibility to nasopharyngeal carcinoma. JOURNAL OF FIRST MILITARY MEDICAL UNIVERSITY 22 (2002) 1126-1127,1130.

[80] E.L. Goode, K.L. White, R.A. Vierkant, C.M. Phelan, J.M. Cunningham, J.M. Schildkraut, A. Berchuck, M.C. Larson, B.L. Fridley, J.E. Olson, P.M. Webb, X. Chen, J. Beesley, G. Chenevix-Trench, T.A. Sellers, C. Ovarian Cancer Association, and G. Australian Ovarian Cancer Study, Xenobiotic-Metabolizing gene polymorphisms and ovarian cancer risk. Mol Carcinog 50 (2011) 397-402.

[81] S.H. Olson, M.D. Carlson, H. Ostrer, S. Harlap, A. Stone, M. Winters, and C.B. Ambrosone, Genetic variants in SOD2, MPO, and NQO1, and risk of ovarian cancer. Gynecol Oncol 93 (2004) 615-20.

[82] G. Huiyin, Association between NQO1 gene polymorphism and risk of endometrial cancer, 2010.

[83] J. Chansaenroj, A. Theamboonlers, P. Junyangdikul, S. Swangvaree, A. Karalak, T. Chinchai, and Y. Poovorawan, Polymorphisms in TP53 (rs1042522), p16 (rs11515 and rs3088440) and NQO1 (rs1800566) genes in Thai cervical cancer patients with HPV 16 infection. Asian Pac J Cancer Prev 14 (2013) 341-6.

[84] K. Nishino, M. Sekine, S. Kodama, N. Sudo, Y. Aoki, N. Seki, and K. Tanaka, Cigarette smoking and glutathione S-transferase M1 polymorphism associated with risk for uterine cervical cancer. J Obstet Gynaecol Res 34 (2008) 994-1001.

[85] Y. Niwa, K. Hirose, T. Nakanishi, A. Nawa, K. Kuzuya, K. Tajima, and N. Hamajima, Association of the NAD(P)H: quinone oxidoreductase C609T polymorphism and the risk of cervical cancer in Japanese subjects. Gynecol Oncol 96 (2005) 423-9.

[86] T. da Rocha Boeira, J. Coser, J.M. Wolf, B.K.M. Cardinal, I. Grivicich, D. Simon, and V.R. Lunge, Polymorphism Located in the Upstream Region of the RPS19 Gene (rs2305809) Is Associated With Cervical Cancer: A Case-control Study. J Cancer Prev 23 (2018) 147-152.

[87] S. Yang, J. Zhao, and L. Li, NAD(P)H: quinone oxidoreductase 1 gene rs1800566 polymorphism increases the risk of cervical cancer in a Chinese Han sample: A STROBE-complaint case-control study. Medicine (Baltimore) 99 (2020) e19941.

[88] C.E. Aston, D.A. Ralph, D.P. Lalo, S. Manjeshwar, B.A. Gramling, D.C. DeFreese, A.D. West, D.E. Branam, L.F. Thompson, M.A. Craft, D.S. Mitchell, C.D. Shimasaki, J.J. Mulvihill, and E.R. Jupe, Oligogenic combinations associated with breast cancer risk in women under 53 years of age. Hum Genet 116 (2005) 208-21.

[89] C.C. Hong, C.B. Ambrosone, J. Ahn, J.Y. Choi, M.L. McCullough, V.L. Stevens, C. Rodriguez, M.J. Thun, and E.E. Calle, Genetic variability in iron-related oxidative stress pathways (Nrf2, NQ01, NOS3, and HO-1), iron intake, and risk of postmenopausal breast cancer. Cancer Epidemiol Biomarkers Prev 16 (2007) 1784-94.

[90] M. Hubackova, R. Vaclavikova, M. Mrhalova, K. Kubackova, R. Kodet, I. Gut, and P. Soucek, NAD(P)H:quinone oxidoreductase 1 Pro187Ser polymorphism and expression do not cosegregate with clinico-pathological characteristics of human mammary tumors. Pharmacogenet Genomics 19 (2009) 505-12.

[91] B. Lajin, A. Alhaj Sakur, and A. Alachkar, Association between polymorphisms in apoptotic genes and susceptibility for developing breast cancer in Syrian women. Breast Cancer Res Treat 138 (2013) 611-9.

[92] H.J. Menzel, J. Sarmanova, P. Soucek, R. Berberich, K. Grünewald, M. Haun, and H.G. Kraft, Association of NQO1 polymorphism with spontaneous breast cancer in two independent populations. Br J Cancer 90 (2004) 1989-94.

[93] J. Sarmanová, S. Sůsová, I. Gut, M. Mrhalová, R. Kodet, J. Adámek, Z. Roth, and P. Soucek, Breast cancer: role of polymorphisms in biotransformation enzymes. Eur J Hum Genet 12 (2004) 848-54.

[94] N. Siegelmann-Danieli, I. Ron, B. Kaufman, B. Uzieli, N. Karminsky, and M. Inbar, [Hormone replacement therapy in breast cancer survivors: the Israeli Society for Clinical Oncology and Radiotherapy policy letter]. Harefuah 141 (2002) 994-6, 1008.

[95] V. Singh, G. Upadhyay, N. Rastogi, K. Singh, and M.P. Singh, Polymorphism of xenobiotic-metabolizing genes and breast cancer susceptibility in North Indian women. Genet Test Mol Biomarkers 15 (2011) 343-9.

[96] M.A. Malik, S.A. Zargar, and B. Mittal, Role of NQO1 609C>T and NQO2-3423G>A polymorphisms in susceptibility to gastric cancer in Kashmir valley. DNA Cell Biol 30 (2011) 297-303.

[97] C.W.R.-f.X.-m.M.-j. Tan-wei, Relationship between NQO1 gene C609T polymorphism and genetic susceptibility and clinicopathological characteristics of breast cancer. Chinese Journal of Clinical and Experimental Pathology 31 (2015) 1243-1247.

[98] O. Nunobiki, M. Ueda, H. Akise, S. Izuma, K. Torii, Y. Okamoto, I. Tanaka, S. Noda, K. Akashi, and T. Higashida, GSTM1, GSTT1, and NQO1 polymorphisms in cervical carcinogenesis. Hum Cell 28 (2015) 109-13.

[99] W.M.-j.R.-f.X.-m.W. Mei-hua, Relationship and significance between C609 T polymorphism of NQO1 gene and breast cancer molecular subtype. Chinese Journal of Clinical and Experimental Pathology (2015) 10-14.

[100] H. Bartsch, C. Malaveille, A.B. Lowenfels, P. Maisonneuve, A. Hautefeuille, and P. Boyle, Genetic polymorphism of N-acetyltransferases, glutathione S-transferase M1 and NAD(P)H:quinone oxidoreductase in relation to malignant and benign pancreatic disease risk. The International Pancreatic Disease Study Group. Eur J Cancer Prev 7 (1998) 215-23.

[101] B. Mohelnikova-Duchonova, L. Marsakova, D. Vrana, I. Holcatova, M. Ryska, Z. Smerhovsky, A. Slamova, M. Schejbalova, and P. Soucek, Superoxide dismutase and nicotinamide adenine dinucleotide phosphate: quinone oxidoreductase polymorphisms and pancreatic cancer risk. Pancreas 40 (2011) 72-8.

[102] F. Liu, L. Luo, Y. Wei, W. Wang, B. Li, L. Yan, and T. Wen, A functional NQO1 609C>T polymorphism and risk of hepatocellular carcinoma in a Chinese population. Tumour Biol 34 (2013) 47-53.

[103] H. Akkiz, S. Bayram, A. Bekar, E. Akgöllü, Y. Ülger, B.Y. Kaya, M. Sandikçi, and B. Özdil, No association of NAD(P)H: quinone oxidoreductase 1 (NQO1) C609T polymorphism and risk of hepatocellular carcinoma development in Turkish subjects. Asian Pac J Cancer Prev 11 (2010) 1051-8.

[104] T.S.-k.X.-q.G.-f.W.-w. Shun, Relationship between hepatocellular carcinoma and the interaction between NQO1 polymorphisms and environmental factors. Chinese Journal of Hepatology 20 (2012) 833-837.

[105] W.W.-w.G.-y.J.-j.G.-f.Z.-t. Han-zong, The relationship between polymorphisms of NQO1 genes and hepatocellular carcinoma in Zhengzhou and Guilin areas. Chinese Journal of Hepatobiliary Surgery 19 (2013) 836-840.

[106] Gui-Wenbo, NQO1 protein expression, NQO1 gene polymorphism and genetic susceptibility to familial aggregated hepatocellular carcinoma, 2011.

[107] Zhang-Wentao, Evaluation of gene polymorphisms associated with gastric cancer susceptibility by Logistic regression and multifactor dimensionality reduction analysis.

Chinese Journal of Biologicals 27 (2014) 679-683.

[108] D.J. Chen, R. Ding, W. Cao, and D.Q. Ye, [Interaction between polymorphisms in NQO1(C609T) and XRCC1(G28152A) and their correlation with smoking on gastric cancer]. Zhonghua Liu Xing Bing Xue Za Zhi 32 (2011) 5-8.

[109] Chen-Daojun, Interaction between NQO1, XRCC1 gene polymorphisms and environmental factors and gastric carcinogenesis, 2007.

[110] W.G. Hu, J.J. Hu, W. Cai, M.H. Zheng, L. Zang, Z.T. Wang, and Z.G. Zhu, The NAD(P)H: quinine oxidoreductase 1 (NQO1) gene 609 C>T polymorphism is associated with gastric cancer risk: evidence from a case-control study and a meta-analysis. Asian Pac J Cancer Prev 15 (2014) 2363-7.

[111] Y. Li, J.H. Zhang, W. Guo, R. Wang, D.G. Wen, and L.Z. Wei, [Polymorphism of NAD(P)H dehydrogenase (quinone) 1 (NQO1) C 609 T and risk of esophageal neoplasm]. Zhonghua Liu Xing Bing Xue Za Zhi 25 (2004) 731.

[112] R.J.-j.X.-h. Xiu-lan, NAD(P)H: quinone oxidoreductase gene polymorphism association with gastric carcinoma. CHINESE JOURNAL OF CANCER PREVENTION AND TREATMENT 13 (2006) 1686-1688.

[113] M. Sarbia, M. Bitzer, D. Siegel, D. Ross, W.A. Schulz, R.B. Zotz, S. Kiel, H. Geddert, Y. Kandemir, A. Walter, R. Willers, and H.E. Gabbert, Association between NAD(P)H: quinone oxidoreductase 1 (NQ01) inactivating C609T polymorphism and adenocarcinoma of the upper gastrointestinal tract. Int J Cancer 107 (2003) 381-6.

[114] J.H. Zhang, Y. Li, R. Wang, H. Geddert, W. Guo, D.G. Wen, Z.F. Chen, L.Z. Wei, G. Kuang, M. He, L.W. Zhang, M.L. Wu, and S.J. Wang, NQO1 C609T polymorphism associated with esophageal cancer and gastric cardiac carcinoma in North China. World journal of gastroenterology 9 (2003) 1390-3.

[115] E. di Martino, L.J. Hardie, C.P. Wild, Y.Y. Gong, J.R. Olliver, M.D. Gough, and N.C. Bird, The NAD(P)H:quinone oxidoreductase I C609T polymorphism modifies the risk of Barrett esophagus and esophageal adenocarcinoma. Genet Med 9 (2007) 341-7.

[116] F.X.-x.Z.-f.L.-b.J.-b. Zu-xun, Study on the relationship between polymorphisms of NQO1 gene and susceptibility to esophageal cancer. CHINESE JOURNAL OF DISEASE CONTROL & PREVENTION 12 (2008) 112-114.

[117] J.J. Freriksen, J. Salomon, H.M. Roelofs, R.H. Te Morsche, J.W. van der Stappen, P. Dura, B.J. Witteman, M. Lacko, and W.H. Peters, Genetic polymorphism 609C>T in NAD(P)H:quinone oxidoreductase 1 enhances the risk of proximal colon cancer. J Hum Genet 59 (2014) 381-6.

[118] J. Yin, L. Wang, X. Wang, L. Zheng, Y. Shi, A. Shao, W. Tang, G. Ding, C. Liu, R. Liu, S. Chen, and H. Gu, NQO1 rs1800566 C>T polymorphism was associated with a decreased risk of esophageal cancer in a Chinese population. Scand J Gastroenterol 49 (2014) 317-22.

[119] L.T.Y.B.M. Chunfeng, Relationship between NQO1 genetic polymorphism and susceptibility of esophageal cancer in Hakkaness. Journal of Modern Oncology 27 (2019) 1911-1915.

[120] M.A. Malik, S.A. Zargar, and B. Mittal, Role of NQO1 609C>T and NQO2 -3423G>A gene polymorphisms in esophageal cancer risk in Kashmir valley and meta analysis. Mol Biol Rep 39 (2012) 9095-104.

[121] H.A. Marjani, F. Biramijamal, N. Rakhshani, A. Hossein-Nezhad, and R. Malekzadeh, Investigation of NQO1 genetic polymorphism, NQO1 gene expression and PAH-DNA adducts in ESCC. A case-control study from Iran. Genet Mol Res 9 (2010) 239-49.

[122] M. Umar, R. Upadhyay, S. Kumar, U.C. Ghoshal, and B. Mittal, Null association of NQO1 609C>T and NQO2 -3423G>A polymorphisms with susceptibility and prognosis of Esophageal cancer in north Indian population and meta-analysis. Cancer Epidemiol 36 (2012) e373-9.

[123] B.H. von Rahden, H.J. Stein, R. Langer, C.W. von Weyhern, E. Schenk, C. Doring, J.R. Siewert, H. Hofler, and M. Sarbia, C609T polymorphism of the NAD(P)H:quinone oxidoreductase I gene does not significantly affect susceptibility for esophageal adenocarcinoma. Int J Cancer 113 (2005) 506-8.

[124] W.C. Zhang, L.H. Yin, Y.P. Pu, G.Y. Liang, X. Hu, Y.Z. Liu, and Y.S. Cui, [Relationship between quinone oxidoreductase1 gene ns-cSNP and genetic susceptibility of esophageal cancer]. Zhonghua Yu Fang Yi Xue Za Zhi 40 (2006) 324-7.

[125] J. Zhang, W.A. Schulz, Y. Li, R. Wang, R. Zotz, D. Wen, D. Siegel, D. Ross, H.E. Gabbert, and M. Sarbia, Association of NAD(P)H: quinone oxidoreductase 1 (NQO1) C609T polymorphism with esophageal squamous cell carcinoma in a German Caucasian and a northern Chinese population. Carcinogenesis 24 (2003) 905-9.

[126] C. Zhouyanli, Shi Xishun, Zhouzijing, Liguoliang, Panpeichuan, Chenzilong, Wuqingbo, A Case-control Study on the Polymorphisms of NQO1 and Susceptibility of Esophageal Cancer. BULLETIN OF CHINESE CANCER 15 (2006) 659-663.

[127] A. Begleiter, D. Hewitt, A.W. Maksymiuk, D.A. Ross, and R.P. Bird, A NAD(P)H:quinone oxidoreductase 1 polymorphism is a risk factor for human colon cancer. Cancer Epidemiol Biomarkers Prev 15 (2006) 2422-6.

[128] Dai-Enyong, The NAD(P)H:Quinone Oxidoreductase 1 Gene Polymorphism and its Association with Colorectal Cancer. CHINESE JOURNAL OF CLINICAL ONCOLOGY 31 (2004) 89-91.

[129] T. Hamachi, O. Tajima, K. Uezono, S. Tabata, H. Abe, K. Ohnaka, and S. Kono, CYP1A1, GSTM1, GSTT1 and NQO1 polymorphisms and colorectal adenomas in Japanese men. World J Gastroenterol 19 (2013) 4023-30.

[130] V. Harth, S. Donat, Y. Ko, J. Abel, H. Vetter, and T. Brüning, NAD(P)H quinone oxidoreductase 1 codon 609 polymorphism and its association to colorectal cancer. Arch Toxicol 73 (2000) 528-31.

[131] I. Hlavata, D. Vrana, Z. Smerhovsky, B. Pardini, A. Naccarati, P. Vodicka, J. Novotny, B. Mohelnikova-Duchonova, and P. Soucek, Association between exposure-relevant polymorphisms in CYP1B1, EPHX1, NQO1, GSTM1, GSTP1 and GSTT1 and risk of colorectal cancer in a Czech population. Oncol Rep 24 (2010) 1347-53.

[132] L. Hou, N. Chatterjee, W.Y. Huang, A. Baccarelli, S. Yadavalli, M. Yeager, R.S. Bresalier, S.J. Chanock, N.E. Caporaso, B.T. Ji, J.L. Weissfeld, and R.B. Hayes, CYP1A1 Val462 and NQO1 Ser187 polymorphisms, cigarette use, and risk for colorectal adenoma. Carcinogenesis 26 (2005) 1122-8.

[133] Lin-Rong, Relationship between NQO1 gene polymorphism, environmental exposure and colorectal cancer susceptibility, 2010.

[134] P.N. Mitrou, M.A. Watson, A.S. Loktionov, C. Cardwell, M.J. Gunter, W.S. Atkin, C.P. Macklin, T. Cecil, D.T. Bishop, J. Primrose, and S.A. Bingham, Role of NQO1C609T and EPHX1 gene polymorphisms in the association of smoking and alcohol with sporadic distal colorectal adenomas: results from the UKFSS Study. Carcinogenesis 28 (2007) 875-82.

[135] H. Nisa, S. Kono, G. Yin, K. Toyomura, J. Nagano, R. Mibu, M. Tanaka, Y. Kakeji, Y. Maehara, T. Okamura, K. Ikejiri, K. Futami, T. Maekawa, Y. Yasunami, K. Takenaka, H. Ichimiya, and R. Terasaka, Cigarette smoking, genetic polymorphisms and colorectal cancer risk: the Fukuoka Colorectal Cancer Study. BMC Cancer 10 (2010) 274.

[136] X.E. Peng, Y.Y. Jiang, X.S. Shi, and Z.J. Hu, NQO1 609C>T polymorphism interaction with tobacco smoking and alcohol drinking increases colorectal cancer risk in a Chinese population. Gene 521 (2013) 105-10.

[137] Peng-Xiane, Relationship between NQO1 C609T gene polymorphism and risk of colorectal cancer. CHINESE JOURNAL OF PUBLIC HEALTH 26 (2010) 415-416.

[138] C. Sachse, G. Smith, M.J. Wilkie, J.H. Barrett, R. Waxman, F. Sullivan, D. Forman, D.T. Bishop, C.R. Wolf, and G. Colorectal Cancer Study, A pharmacogenetic study to investigate the role of dietary carcinogens in the etiology of colorectal cancer. Carcinogenesis 23 (2002) 1839-49.

[139] A.S. Sameer, Z.A. Shah, N. Syeed, R. Rasool, D. Afroze, and M.A. Siddiqi, NAD(P)H:quinone oxidoreductase 1 (NQO1) Pro187Ser polymorphism and colorectal cancer predisposition in the ethnic Kashmiri population. Asian Pac J Cancer Prev 11 (2010) 209-13.

[140] X.L. Su, M.R. Yan, L. Yang, and S. Qimuge, NQO1 C609T polymorphism correlated to colon cancer risk in farmers from western region of Inner Mongolia. Chin J Cancer Res 24 (2012) 317-22.

[141] M.J. Tijhuis, M.H. Visker, J.M. Aarts, W. Laan, S.Y. de Boer, F.J. Kok, and E. Kampman, NQO1 and NFE2L2 polymorphisms, fruit and vegetable intake and smoking and the risk of colorectal adenomas in an endoscopy-based population. Int J Cancer 122 (2008) 1842-8.

[142] E.M. van der Logt, S.M. Bergevoet, H.M. Roelofs, R.H. Te Morsche, Y. Dijk, T. Wobbes, F.M. Nagengast, and W.H. Peters, Role of epoxide hydrolase, NAD(P)H:quinone oxidoreductase, cytochrome P450 2E1 or alcohol dehydrogenase genotypes in susceptibility to colorectal cancer. Mutat Res 593 (2006) 39-49.

[143] Liu-Xiyong, Relationship between polymorphisms of NAT2, NQ01 and MTHFR of metabolic enzyme genes and recurrence of rectal adenoma, 2002.

[144] Li-Yan, NAD (P) h: quinone oxidoreductase 1 C609T polymorphism and risk of cardiac cancer. CHINESE JOURNAL OF EPIDEMIOLOGY 25 (2004) 731.
