# Supplementary figures and images for: Research on the effects of rs1800566 C/T polymorphism of NAD(P)H quinone oxidoreductase 1 gene on cancer risk involves analysis of 43,736 cancer cases and 56,173 controls
Source: Front Oncol. 2022 Oct 19;12:980897. doi: 10.3389/fonc.2022.980897 (PMC9627178; doi:10.3389/fonc.2022.980897)

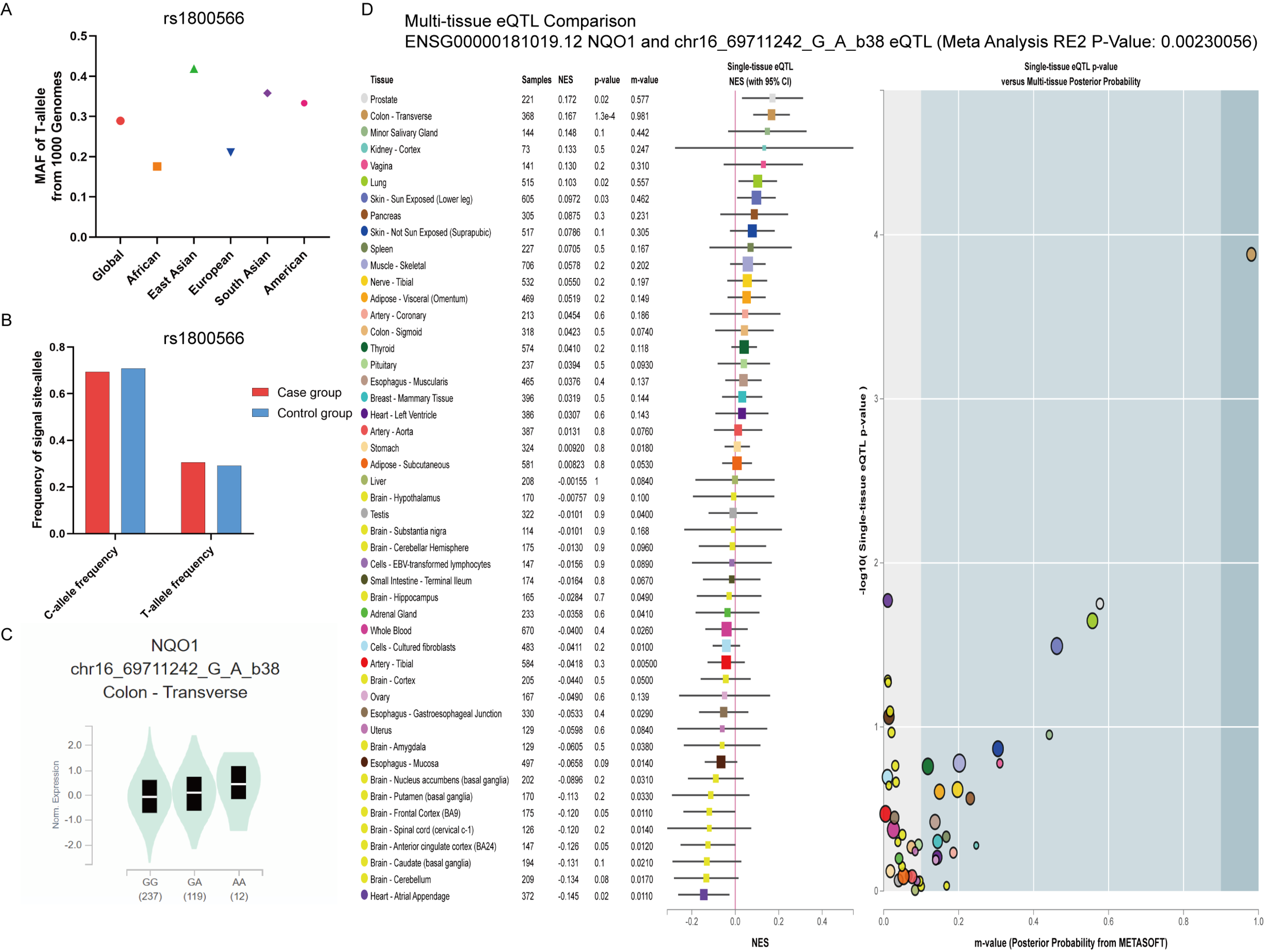

Supplement: Supplementary Figure 1 — (A) The MAF for the NQO1 rs1800566 polymorphism from the online 1000 Genome. (B) C- and T-allele frequencies in the case and control groups. (C) The genotype distribution from the GTEx Portal website (the site’s main page is located at https://www.gtexportal.org). (D) The risk frequency of rs1800566 polymorphism in many types of diseases from TCGA database. [file Image_1.tif]
